# Towards plant resistance to viruses using protein-only RNase P

**DOI:** 10.1038/s41467-021-21338-6

**Published:** 2021-02-12

**Authors:** Anthony Gobert, Yifat Quan, Mathilde Arrivé, Florent Waltz, Nathalie Da Silva, Lucile Jomat, Mathias Cohen, Isabelle Jupin, Philippe Giegé

**Affiliations:** 1 Institut de biologie moléculaire des plantes, UPR2357 du CNRS, Université de Strasbourg, Strasbourg, France; 2 Institut Jacques Monod, Laboratory of Molecular Virology, UMR7592 CNRS, Université de Paris, Paris, France

**Keywords:** Nucleases, RNA-binding proteins, Genetic engineering, Molecular engineering in plants

## Abstract

Plant viruses cause massive crop yield loss worldwide. Most plant viruses are RNA viruses, many of which contain a functional tRNA-like structure. RNase P has the enzymatic activity to catalyze the 5′ maturation of precursor tRNAs. It is also able to cleave tRNA-like structures. However, RNase P enzymes only accumulate in the nucleus, mitochondria, and chloroplasts rather than cytosol where virus replication takes place. Here, we report a biotechnology strategy based on the re-localization of plant protein-only RNase P to the cytosol (CytoRP) to target plant viruses tRNA-like structures and thus hamper virus replication. We demonstrate the cytosol localization of protein-only RNase P in Arabidopsis protoplasts. In addition, we provide in vitro evidences for CytoRP to cleave turnip yellow mosaic virus and oilseed rape mosaic virus. However, we observe varied in vivo results. The possible reasons have been discussed. Overall, the results provided here show the potential of using CytoRP for combating some plant viral diseases.

## Abstract (web summary)

New approaches to plant disease control are important for pathogens that are difficult to control by existing methods. Here, the authors report a potential strategy to combat plant viruses by cytosolic expressed protein-only RNase P and show its ability for in vitro cleavage of tRNA-like structures existing in many plant viruses.

## Introduction

One of the major challenges of the 21st century is to produce sufficient food resources for a rapidly growing humanity, while the availability of cultivated land is decreasing and the environment is changing. Currently, about 900 million people suffer from chronic malnutrition, and in this context, the increase of agricultural yields will be decisive. Plant viruses are among the most devastating pathogens, being responsible for significant damages on most agronomically important crops, estimated at tens of billions U.S. dollars annually^1^, an importance that may yet increase since viruses constitute the largest cohort of emerging crop diseases^2^. The development of biotechnologies for the control of viral diseases is thus required to sustain food production.

More than 80% of plant viruses have an RNA genome and a significant number of them bear a tRNA-like structure (TLS) at the 3′ end of their genomic RNA, which is critical for viral infectivity and for virus replication^3–6^. Viruses containing TLS, including many widespread viruses such as cucumber mosaic virus (CMV), oilseed rape mosaic virus (ORMV), and its close relative tobacco mosaic virus (TMV) or turnip yellow mosaic virus (TYMV) (Supplementary Table 1), infect a wide range of crops of major economic importance worldwide such as wheat, corn, beans, tomato, cabbage, pepper, cucurbits, potato, peanut, or cocoa trees.

RNase P is an endonuclease activity responsible for one of the key steps of tRNA precursors maturation. It removes 5′ leader sequences of pre-tRNAs^7^ and is thus essential to obtain functional tRNAs required for translation. Until recently, all characterized RNase P enzymes were ribonucleoproteins, containing an RNA holding catalytic activity^7^. However, another type of RNase P only composed of protein was discovered in eukaryotes^8^. Beyond tRNA maturation, these enzymes called PRORP also participate in mRNA maturation by cleaving TLS present at the 5′ or 3′ termini of some plant mitochondria mRNAs, as shown in Arabidopsis both in vitro and in vivo^9,10^.

**Fig. 1:**
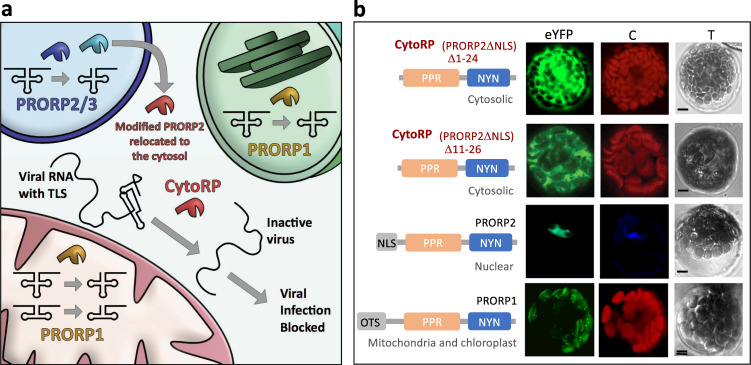
Strategy to make Arabidopsis cells resistant to viruses containing TLS by directing an RNase P enzyme—referred as CytoRP—to the cytosol. **a** Principle of the CytoRP technology showing that endogenous plant PRORP enzymes localizations are restricted to mitochondria, chloroplasts, and the nucleus. Because the two PRORP enzymes which are present in Arabidopsis nuclei, have redundant functions^10^, it can be envisaged to relocate one of them (PRORP2) to the cytosol, where virus RNA translation/replication takes place. Nuclear PRORP proteins are shown in blue, organellar PRORP1 in yellow, and the cytosolic CytoRP in red. **b** CytoRP proteins obtained by two different deletions, i.e. of the first 24 amino acids or of residues 11–26, accumulate in the cytosol while PRORP2 and PRORP1 accumulate in the nucleus and organelles, respectively. NLS and OTS show the nuclear localization signal and organellar targeting sequences localized upstream of the functional PPR and NYN domains of PRORP enzymes. The subcellular localization of CytoRP and PRORP protein was analyzed by confocal microscopy of proteins fused to eYFP. Independent observations were performed three times. C indicates the autofluorescence of chlorophyll for CytoRP as well as PRORP1 and DAPI staining for PRORP2. T are transmitted light images of Arabidopsis protoplasts transformed with the respective constructs. Scale bars correspond to 5 μm. Source data underlying **b** are provided as a Source Data file.

Based on this observation, we report here a strategy to induce plant resistance to viruses containing a TLS. We rationalize that an RNase P enzyme should be able to cleave TLS of plant viruses, which would impede virus replication and thus impair virus infection. However, such a process is unlikely to occur spontaneously in vivo because RNA virus infection takes place in the cell cytosol^11^, while PRORP enzymes accumulate only in compartments where endogenous gene expression takes place, i.e. the nucleus, mitochondria, and chloroplasts^9^. We thus develop an approach to allow RNase P enzymes to accumulate in the cytosol. Early work had already shown that bacterial RNA-based RNase P is able to cleave the TLS of plant viruses, in particular that of the TYMV^12^. However, the manipulation of RNase P ribozyme localization is hardly feasible, and we thus modify a plant PRORP enzyme to allow its accumulation in the cytosol (Fig. 1a). Both Arabidopsis PRORP enzymes and the TYMV TLS were previously well characterized at the functional and structural levels^13,14^. We use the model pathosystems *Arabidopsis thaliana* and TYMV or ORMV to evaluate this strategy both in vitro and in vivo. The PRORP enzyme re-located to the cytosol, called CytoRP for cytosolic RNase P, is able to cleave TYMV and ORMV TLS in vitro. The effect of CytoRP expression on virus levels upon infection is also evaluated in planta.

## Results

### Design and cytosolic localization of CytoRP proteins

PRORP enzymes contain two main domains, an N-terminal pentatricopeptide repeats (PPR) domain responsible for substrate-specific RNA binding, and a C-terminal Nedd4-BP1, YacP nuclease (NYN) domain that holds the endonuclease activity^15^. In Arabidopsis, three PRORP enzymes have been described: PRORP1 accumulates in both mitochondria and chloroplasts while PRORP2 and PRORP3 are both found in the nucleus^9^. Because previous work had suggested that the latter have redundant functions^10^, we rationalized that one nuclear PRORP could be re-localized to the cytosol. For this, a stretch of positively charged residues predicted to constitute a nuclear localization signal (NLS) conserved in land plants nuclear PRORP enzymes (Supplementary Fig. 1) was deleted from PRORP2, creating truncated proteins called CytoRP. Two versions of CytoRP were generated: (i) with a deletion of amino-acids 1–24 and (ii) with a deletion of amino-acids 11–26, corresponding to the sequence obtained in CytoRP plants generated by genome editing (see below). Despite the removal of these sequences, CytoRP proteins retain all the elements necessary for RNase P activity, i.e. the PPR and the NYN domains. When fused to eYFP and expressed in plant protoplasts, fluorescence microscopy experiments showed that CytoRP-eYFP constructs were indeed relocated to the cytosol, while control PRORP2-eYFP and PRORP1-eYFP accumulated in the nucleus or mitochondria/chloroplasts, respectively (Fig. 1b).

### CytoRP is able to cleave different virus TLSs in vitro

**Fig. 2:**
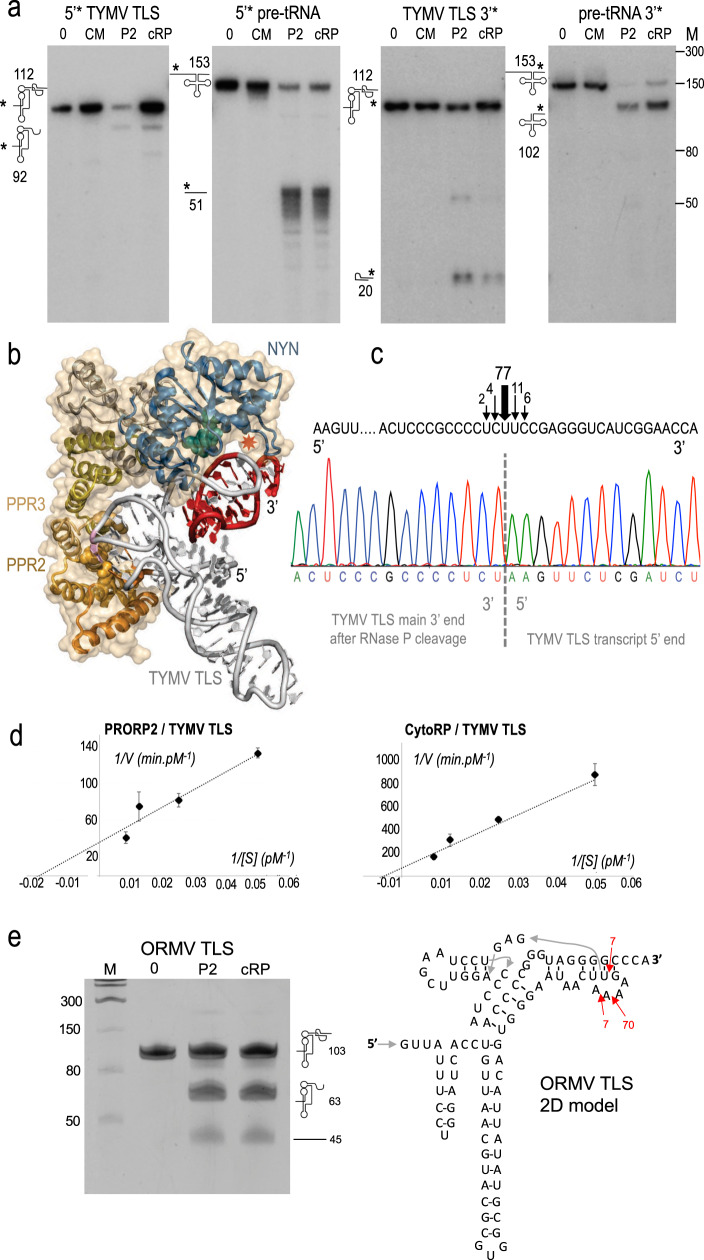
CytoRP is able to cleave the TYMV and ORMV TLS in vitro. **a** In vitro RNase P activity assays performed with transcripts corresponding to the TYMV TLS and Arabidopsis pre-tRNA^Cys^ labeled in 5′ or 3′ as well as CytoRP proteins (cRP), wild type PRORP2 (P2) and a catalytically inactive mutant (CM) as described in the “Methods” section. 0 indicates lanes where no protein was added to the reactions. Numbers on the left indicate the calculated sizes of RNA fragments expected to result from canonical RNase P activity. M shows single-stranded RNA molecular weight markers in nucleotides. The TYMV transcript is 112 nt long, with the final 83 nt being the TLS. RNase P cleavage results in a 92 nt long 5′ product and a 20 nt long 3′ product. Cleavages were performed in at least three independent experiments. **b** Tridimensional model of CytoRP interaction with the TYMV TLS based on PRORP2 crystal structure^13^, the TYMV TLS crystal structure^14^ and both X-ray crystallography and SAXS data on the complex formed by PRORP2 and tRNA^13,27^. The red star shows the position expected to be cleaved by canonical RNase P activity, close to catalytic aspartates (blue spheres) of the NYN domain. **c** Precise mapping of the TLS cleavage site by CytoRP was performed by circular RT-PCR of the 5′-cleavage product, cloning, and sequencing. The chromatogram shows the sequence of a representative clone. Arrows and numbers indicate the position and percentage of clones among the 53 clones analyzed resulting from cleavage events. 77% of them took place at the position indicated by the red star in Fig. 2b. **d** Kinetic analysis of TYMY TLS cleavage by PRORP2 and CytoRP. Experiments were performed with 1 μM protein for 9 reaction times. Experiments were performed with 20, 40, 80, and 120 pM of RNA substrate. Average values from triplicate experiments are shown in a Lineweaver–Burk plot that was used to derive *K*_m_ and *V*_max_ values. Error bars represent the standard deviation for three replicate experiments. **e** In vitro RNase P activity assays performed with a transcript corresponding to the ORMV TLS as described for TYMV on **a**. Numbers on the right indicate the calculated sizes of RNA fragments expected to result from RNase P activity according to a predicted 2D fold of the ORMV TLS presented on the right. Precise mapping of the TLS cleavage site by CytoRP was performed by circular RT-PCR of the 5′-cleavage product, cloning and sequencing. The positions of cleavage sites are indicated on the 2D model, with red arrows and numbers indicate the position and percentage of clones among the 113 clones analyzed resulting from cleavage events (for positions >5%). Source data underlying **a**, **d**, and **e** are provided as a Source Data file.

As a next step, recombinant CytoRP (with the 1–24 deletion) was expressed in *E. coli*, affinity-purified (Supplementary Fig. 2) and used for in vitro RNase P activity assays. As a control, a catalytically inactive enzyme was also used, in which two conserved aspartate residues of the NYN domain, previously shown to be essential for RNase P activity^9^, were substituted to alanines. CytoRP (cRP), its catalytically inactive mutant (CM) as well as wild type PRORP2 (P2) were then used in activity assays to test their ability to cleave the TLS of in vitro transcripts corresponding to the 3′ end of TYMV genomic RNA. Results using both 5- and 3′-end radiolabelled transcripts showed that CytoRP is able to cleave the TYMV TLS in vitro, as cleavage products with sizes matching those expected to result from canonical RNase P activity were detected in lanes containing CytoRP (Fig. 2a). As a positive control, activity assays were also performed using an Arabidopsis endogenous tRNA precursor as substrate.

In order to determine the exact cleavage position(s) within TYMV TLS, the 5′ cleavage product of the TLS was then characterized by circular RT-PCR, cloning, and sequencing. This revealed that the majority of cleavage events had taken place in the TLS pseudoknot, 20 nt upstream of the RNA 3′ end. This indicates that CytoRP performed canonical RNase P activity and cleaved the TLS at the position predicted from the analysis of a three-dimensional model of the CytoRP/TLS complex (Fig. 2b, c), and similar to bacterial ribozyme RNase P^12^. This result thus demonstrates that CytoRP recognition and binding of the 3D fold of the TYMV TLS is similar to that of PRORP2 recognition of a pre-tRNA^13^. Minor cleavages also occurred 1 or 2 nt upstream or downstream of the main cleavage site, thus suggesting a flexibility of the TYMV TLS 3D fold in vitro. In order to better characterize the cleavage of the TYMV TLS by CytoRP, kinetic analyses were also performed in single turnover conditions, revealing a *K*_m_ of 222 × 10^−6^ μM and a *V*_max_ of 2.4 × 10^−7^ nM/s, whereas wild type PRORP2 cleaved the TYMV TLS with a *K*_m_ of 52 × 10^−6^ μM and a *V*_max_ of 4.6 × 10^−7^ nM/s, thus showing that PRORP2 is a somewhat better catalyst in vitro than its mutant CytoRP (Fig. 2d, Supplementary Fig. 3).

Then, in order to show that the CytoRP strategy could be applicable to different viruses, a transcript representing the 3′ end of ORMV genomic RNA composed of a TLS was used for RNase P activity assays. RNA cleavage was observed and precisely mapped by circular RT-PCR. This showed that most cleavages occurred in a predicted pseudoknot of the TLS, 43 nt upstream of the RNA 3′ end (Fig. 2e). Altogether results showed that CytoRP can process TLS structures from different viruses in vitro.

### Assessment of CytoRP strategy in vivo

In order to assess whether CytoRP affects TYMV and ORMV accumulation in vivo, i.e. whether viral RNA accumulation could be decreased in planta, we then created a series of plants expressing CytoRP. Transgenic plant lines were constructed by classical Agrobacterium-mediated transformation with the CytoRP gene, containing the 1–24 deletion, expressed either under control of the endogenous PRORP2 promoter or the strong constitutive 35S promoter of *Cauliflower mosaic virus*. In addition, cisgenic plant lines were also generated by genome editing through the deletion of 16 amino acids of PRORP2 starting at the 11th amino acid using CRISPR-Cas9 technology (Supplementary Fig. 4). Six independent transgenic and one cisgenic CytoRP plant lines were further analyzed.

PRORP functions in vivo appear to be strictly restricted to nucleus, mitochondria, and chloroplasts^10^, where they are involved in pivotal gene expression pathways. In this context, re-targeting one of the PRORP isoforms to the cytosol might have caused deleterious effects. However, careful monitoring of CytoRP plant growth and development did not reveal any detrimental effects. The different CytoRP plant lines as well as control Col-0 wild type Arabidopsis plants were subjected to TYMV and ORMV infection. Virus load was monitored 28 days and 7 days, respectively, post-inoculation by measurement of TYMV genomic RNA accumulation by quantitative RT-PCR as described previously^16^. While no macroscopic differences in infection symptoms could be observed between the different lines, CytoRP lines could show statistically significant (assessed by Wilcoxon tests) reductions of both TYMV and ORMV RNA content as compared to wild type plants in some sets of experiments (Supplementary Figs. 5–7). However, viral RNA levels observed in vivo were altogether variable, between replicate experiments and even between individual plants in the same genotypes (Supplementary Figs. 5–7) and no correlation was observed between CytoRP expression levels (Supplementary Fig. 8) and the accumulation of viral RNAs. Altogether, these results show that CytoRP proteins are able to cleave TYMV and ORMV TLS and that their accumulation in the cytosol might result in decreased viral RNA levels upon TYMV and ORMV infection.

## Discussion

Despite the variability that we observed for in vivo results and even if the infected individual plants did not show weaker symptoms upon expression of CytoRP, the anticipated reduced viral RNA levels can dampen the spread of virus within crop plant populations. Still, the CytoRP strategy could be improved to increase its efficiency. For this, CytoRP might be further fused to a specific domain that was shown to target proteins to viral replication complexes in the cytosol at the surface of plastids^17^. Alternatively, further mutations, i.e. to the RNA-binding domain of CytoRP could be envisaged to increase CytoRP affinity to viral TLS as compared to endogenous tRNA precursors.

In all cases, what differentiates the CytoRP antiviral strategy from current antiviral approaches is that it should theoretically allow disease management of many viruses at once, i.e. of all viruses harboring a TLS in their genome (Supplementary Table 1), experimentally assessed here for both the TYMV and ORMV, whereas other strategies target only one particular virus. Indeed, engineered antiviral approaches developed to date are essentially based on pathogen-derived resistance strategies whereby a sequence or part of the viral genome is introduced into the host plant^18^. Resistance may occur either through constitutively (over)expressing a viral protein that may interfere with the viral life cycle due to its imbalanced and/or uncoordinated expression in the host cell or through RNA silencing^19^. Available approaches include expression in plants of artificial microRNAs^20^ or the expression of plantibodies, i.e. recombinant antibodies raised against viral proteins^21^. These approaches are sequence-specific or epitope-specific and thus result in narrow ranges of antiviral resistance.

Another issue of pathogen-derived resistance strategies is the fact that plants genetically engineered for viral resistance encode parts of viral genomes. The risk of interactions between the transgene and the challenging virus—which may cause the appearance of more virulent strains through recombination or trans-encapsidation, is thus a concern for biosafety, human health, or environmental impact^22^. Such a risk is not expected with the CytoRP technology, as it does not depend on the insertion of pathogen-derived sequences into the plant genome.

Furthermore, it can be expected that the chance for a resistance-breaking variant to arise in the viral population is low, because the RNase P cleavage sites in TLS, e.g. that of TYMV or ORMV, are present in pseudoknots (Fig. 2b, e). Structural constraints to maintain the functional RNA-fold might thus prevent evolution of the viral genome. Altogether, the CytoRP approach might have the potential to constitute a source of efficient, broad, and durable resistance against viruses. We thus think that CytoRP technology might bring a valuable potential to the improvement of crop protection and production of safer products, two major societal concerns.

## Methods

### CytoRP design, protein expression, and purification

CytoRP corresponds to Arabidopsis PRORP2 amino acids 25–519 amplified with oligonucleotides CRP5′ and CRP3′. All oligonucleotides sequences used here and mentioned hereafter in the “Methods” section are available in Supplementary Table 2. The CM has NYN active sites Aspartates 421 and 422 mutated to Alanines. PRORP2 cDNAs were cloned upstream of a polyhistidine affinity tag sequence in pET28-b(+) (Novagen). Proteins expression was carried out in *E. coli* BL21(DE3 cells) at 18 °C overnight with 1 mM IPTG for expression induction. Bacteria were lysed and pelleted 30 min at 20,000×*g* 4 °C. Cleared lysates were incubated with the Ni-NTA resin (Qiagen). Proteins retained were washed with buffers including 50 mM imidazole, 20 mM MOPS pH 7.8, 150 mM NaCl and 10% (v/v) glycerol, and 75 mM imidazole, 20 mM MOPS pH 7.8, 250 mM NaCl and 10% (v/v) glycerol. Protein elution was carried out with buffers containing 200 mM imidazole and 500 mM imidazole. Proteins were further purified by size-exclusion chromatography, with a Superdex 200 10/300 Increase column, using an automated Äkta pure system in buffer containing 20 mM MOPS pH 7.8, 150 mM NaCl, and 10% (v/v) glycerol.

### TYMV and ORMV TLS, cloning and transcription

A cDNA fragment representing the 3′ end of TYMV genomic RNA was amplified with oligonucleotides TYMV5′ and TYMV3′, as well as the 3′ end of ORMV genomic RNA was amplified with oligonucleotides ORMV5′ and ORMV3′ containing the *Bam*HI, T7 promoter sequence, and *Eco*RI restriction sites, respectively. The PCR product was cloned in pUC19. The clone used to express an Arabidopsis pre-tRNA Cys was used by Gobert et al. ^9^. Linearized plasmid DNA (200 ng) was transcribed in a 10 µL reaction comprising 7.5 mM rNTP, 5 U T7 RNA polymerase and buffer supplied with the enzyme (TranscriptAid, Thermo Scientific) for 4 h at 37 °C. Plasmid DNA was then digested with 1 U of DNase I (Thermo Scientific) for 15 min at 37 °C and transcripts were cleaned by phenol chloroform (v/v) extractions. RNAs were dephosphorylated with 1 U FastAP alkaline phosphatase (Thermo Scientific) for 30 min at 37 °C and where then either 5′ radiolabelled with ^32^P-γATP and polynucleotide kinase, or 3′ radiolabeled with ^32^P-pCp and T4 RNA ligase (New England Biolabs).

### RNase P activity assays

Cleavage reactions were performed in a 10 µL volume containing 100 ng proteins and 100 ng 5′ or 3′ radiolabelled transcript, in buffer containing 20 mM Tris–HCl pH 8, 30 mM KCl, 4.5 mM MgCl_2_, 20 µg/mL BSA, and 2 mM DTT, at room temperature for 15 min. RNA molecules were separated on 12% polyacrylamide urea gels and visualized by autoradiography and/or by ethidium bromide staining under UV light. For kinetic analyses, cleavages were performed with 1 μM CytoRP or PRORP2, 20, 40, 80, and 120 pM of TYMV TLS RNA substrate for times varying from 5 min to 2 h, in triplicates for each condition.

### Circular RT-PCR analysis

TYMV and ORMV 5′ RNA fragments generated from RNase P cleavages were visualized by Ethidium bromide staining, purified from gel and incubated with 5 U of T4 RNA ligase (New England Biolabs) and 20 U RNase inhibitor (RNase OUT, Invitrogen) in a reaction volume of 25 µL. Circular RNA was used for cDNA synthesis with Super-script III (Invitrogen), with primers CRTTYMV5′ and CRTTYMV3′ for TYMV as well as CRTORMV5′ and CRTORMV3′ for ORMV.

### Construction of CytoRP transgenic plant lines

A PCR-amplified fragment containing the CytoRP cDNA in frame with an HA tag, inserted after either the promoter sequence of Arabidopsis PRORP2 or the CaMV 35S promoter, was cloned in a binary vector and used to generate transgenic plants using Agrobacterium-mediated transformation, by floral dip with *Agrobacterium tumefaciens* GV3101. Plants were selected for hygromycin resistance, by genotyping with primers GtTg5′ and GtTg3′. Plants were also selected for CytoRP expression. The selected plants contain the three PRORP expressed from endogenous genes, as well as the additional CytoRP expressed from the transgene and localized in the cytosol. Six distinct Arabidopsis lines (4 with PRORP2 endogenous promoter and 2 with the 35S promoter), resulting from different insertions of the CytoRP gene randomly in the genome were selected for further use.

### Construction of a CytoRP cisgenic plant lines

A guide RNA targeting the conserved lysines (underlined codons) of PRORP2 NLS was designed AAGACCCAACAAGAAGAAGA. It was cloned into pENchimera entry vector and transferred to pDECas9^23^. Transgenic plants expressing Cas9 and the guide RNA were produced using Agrobacterium-mediated transformation. Deletion were selected by PCR in the T2 generation with primers GtCg5′ and GtCg3′. The selected plant was backcrossed three times with WT Col-0 in order to get rid of the T-DNA insertion and possible off-targets mutations.

### Localization of CytoRP-eYFP fusion proteins

CytoRP cDNA was inserted into the pART7EYFP vector. Arabidopsis mesophyll protoplasts were isolated and transformed with pART7PRORPEYFP plasmids using polyethylene glycol-mediated transformation. EYFP fluorescence was observed by confocal laser scanning microscopy using a Zeiss LSM700 based on an Axiovert 200M microscope (Zeiss).

### Inoculation of Arabidopsis plants

*A. thaliana* plants were grown at 19–21 °C under a 16 h light/8 h dark photoperiod in a growth chamber, and plants were inoculated ~5–6 weeks after seed germination, before the occurrence of floral transition. The inoculum consisted of TYMV and ORMV viral RNA (4 µg per plant) diluted in 45 µL of water. Plants were mechanically inoculated by rubbing the upper surface of three rosette leaves using celite as an abrasive^24^. After inoculation, the plants were allowed to grow in the same conditions, harvested at 28 dpi for TYMV and 7 dpi for ORMV, frozen in liquid nitrogen and stored at –80 °C. A total of 17–20 plants was inoculated in two independent experiments.

### RNA isolation, cDNA synthesis, and quantification of viral RNA accumulation by qPCR

Total RNA extraction was performed using Nucleospin RNA kit (Macherey Nagel) and cDNA synthesis was performed with SuperScript III reverse transcriptase (ThermoFisher). The abundance of viral RNAs in plant samples was performed by quantitative RT PCR, mainly using a Roche LightCycler 480 with EF1α and 18S rRNA as reference genes. Data relative to CytoRP plants were then expressed as a percentage of the mean value of the data obtained with Col-0 plants that were inoculated simultaneously and analyzed by qPCR in the same run. Data are represented as box-plots using BoxPlotR (http://boxplot.tyerslab.com/).

### Bioinformatic analyses

Subcellular localization predictions were determined with SUBA4^25^. Protein similarities were analyzed through MUSCLE Alignments. Tridimensional predictions were built with Phyre2 (http://www.sbg.bio.ic.ac.uk/phyre2) and molecular representations were prepared with PyMol^26^.

### Reporting summary

Further information on research design is available in the Nature Research Reporting Summary linked to this article.

## Supplementary information

Supplementary Information File

Reporting Summary

## Data Availability

Data supporting the findings of this work is available within the paper and its Supplementary Information files. A reporting summary for this Article is available as a Supplementary Information file. The datasets, clones, and plant materials generated and analyzed during the current study are available from the corresponding authors upon request.  Source data are provided with this paper.

## Source data

Source Data
