# Supplementary material for: Towards plant resistance to viruses using protein-only RNase P
Source: Nat Commun. 2021 Feb 12;12:1007. doi: 10.1038/s41467-021-21338-6 (PMC7881203; doi:10.1038/s41467-021-21338-6)
Supplement: Supplementary file 1 — Supplementary Information File [file 41467_2021_21338_MOESM1_ESM.pdf]

Towards plant resistance to viruses using protein-only RNase P

Gobert *et al.*

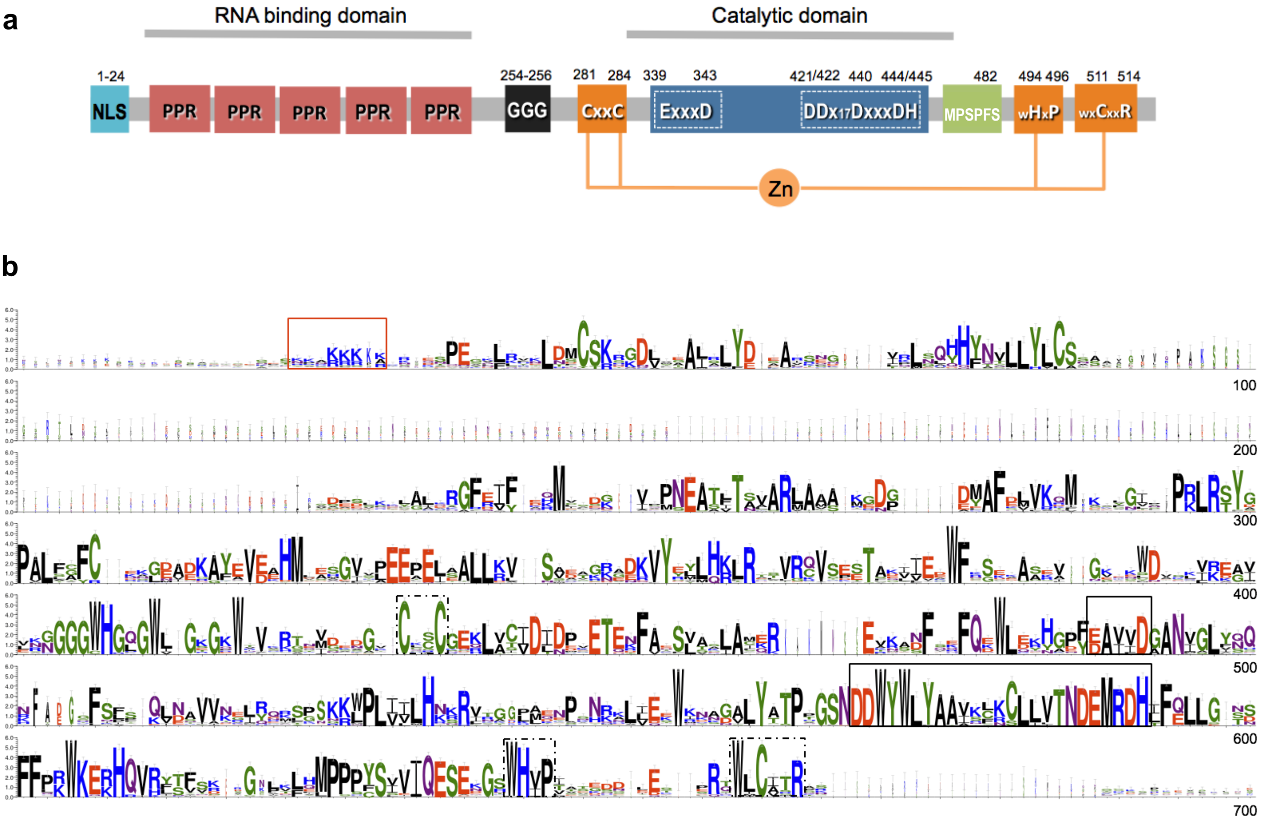

Supplementary Figure 1 Features defining PRORP enzymes. (**a**) Schematic representation of a PRORP enzyme with residues predicted to play an important role highlighted and numbered according to *Arabidopsis* PRORP2 sequence. (**b**) Logos representing residue frequency at each position in an alignment of 91 plant PRORP sequences. Boxed motifs correspond to motifs highlighted in A. In particular, a lysine rich segment at the N-terminal part of proteins (highlighted in red) is conserved in plants and predicted by SUBA4 to confer nuclear localization.

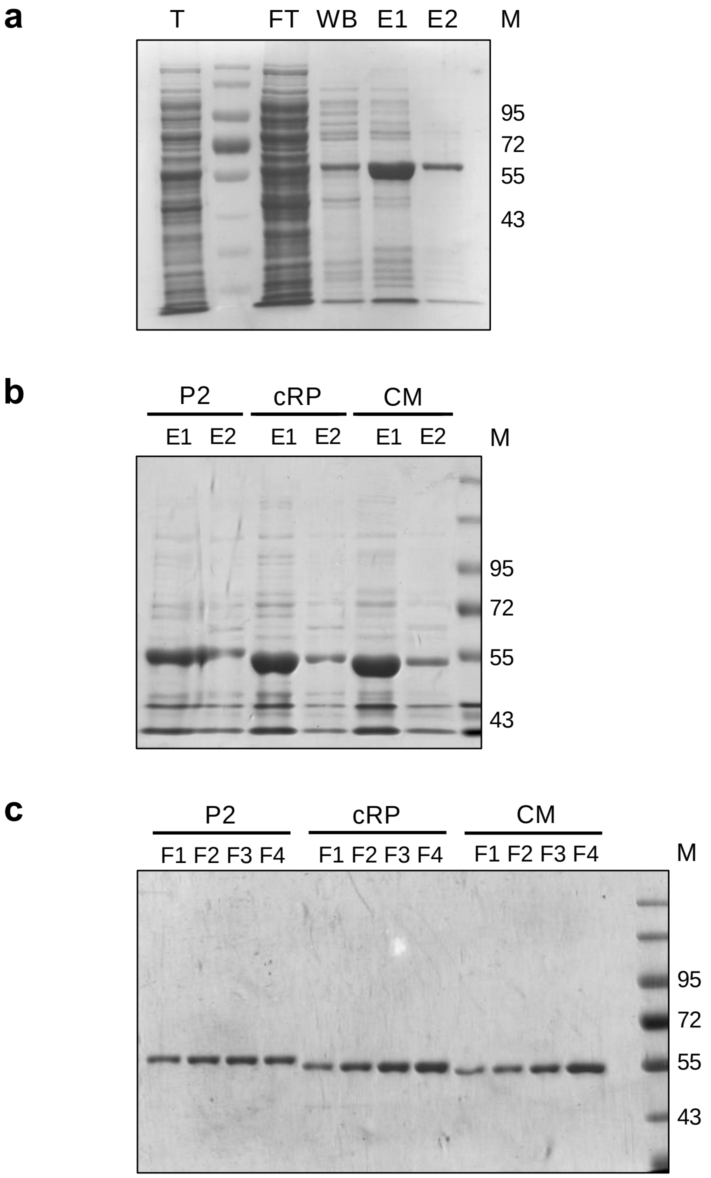

Supplementary Figure 2 Expression and purification of recombinant CytoRP (cRP), its catalytically inactive mutant (CM) and wild type PRORP2 (P2). (**a**) Fractions from a representative CytoRP protein purification by affinity to a poly-histidine tag. T is the total *E. coli* soluble protein extract, FT the Ni affinity column flow through, WB the wash buffer, E1 proteins eluted with buffer containing 200 mM imidazole and E2 proteins eluted with buffer containing 500 mM imidazole. (**b**) Representative elution fractions from CytoRP, its catalytically inactive mutant and PRORP2 purified by affinity chromatography. (**c**) Further purification of the three proteins by size exclusion chromatography; the four major elution fractions (F1-F4) are shown for each protein. Protein samples were separated on 8% SDS-PAGE and gels were stained with Coomassie Blue. Molecular weight markers (M) are indicated in kDa. Protein purification steps shown here in a, b and c were performed several times (n>10), i.e. freshly prepared proteins were always used for cleavage assays shown in Fig. 2a, d and e.

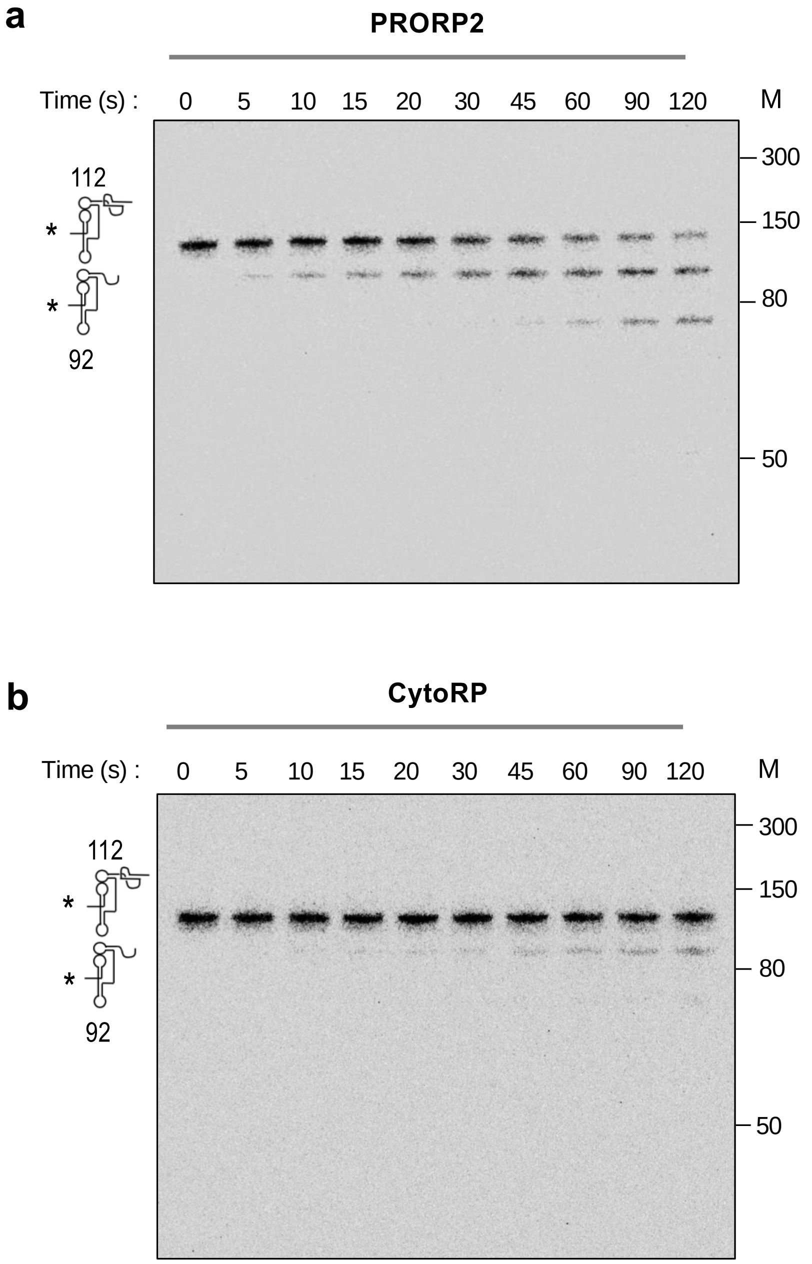

Supplementary Figure 3 Kinetic analyses of TYMY TLS cleavage by PRORP2 **(a)** and CytoRP **(b)**. Experiments were performed with 1 ∝M protein for 9 reaction times indicated here in seconds. Representative gels are shown here for experiments performed with 20 pM of RNA substrate. Other time course experiments were performed with 40, 80 and 120 pM RNA. Each cleavage was performed in triplicate experiments. *In vitro* RNase P activity assays were performed with transcripts corresponding to the TYMV TLS labelled in 5'. The lower molecular weight product (70 nt) corresponds to a secondary cleavage product of the first RNase P cleavage product of the ORMV TLS transcript, that we could characterise by circular RT-PCR. Reactions were analysed on 12% denaturing acrylamide gels, dried and autoradiographed for 2h. Numbers on the left indicate the calculated sizes of RNA fragments expected to result from canonical RNase P activity. M shows molecular weight markers in nucleotides.

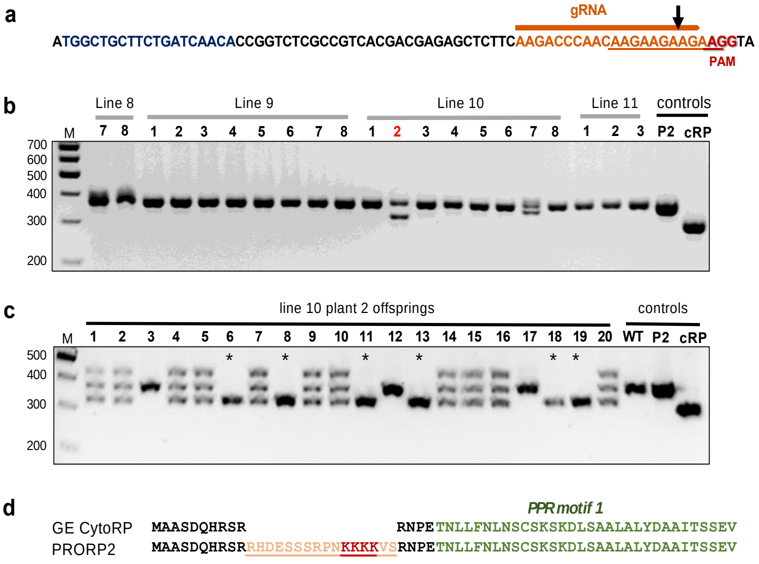

Supplementary Figure 4 Design and identification of genome-edited Arabidopsis plants expressing CytoRP. (**a**) Nucleotide sequence of the 5' end of the endogenous PRORP gene showing the position of the guide RNA (gRNA, orange bar) used for genome editing and the protospacer adjacent motif (PAM). The Cas9 predicted cleavage site is indicated by a black arrow. The underlined sequence codes for the 4 lysine residues conserved across plants. (**b**) Individual plants from 4 independent lines, obtained after transformation with constructs expressing Cas9 and the guide RNA, were genotyped by PCR. Plant 2 from line 10 showed a heterozygous deletion of PRORP2 sequence. P2 and cRP show control PCR reactions performed with cDNAs representing wild type PRORP2 and a CytoRP with a 72 bp deletion. (**c**) The analysis of line 10 plant 2 offsprings identified plants homozygous for a PRORP2 sequence deletion, shown by asterisks. Sequencing revealed that these plants had a 48 nucleotides deletion. This showed that the deletion could be transmitted to the next generation. M show molecular weight markers indicated in nucleotides (**d**) The genome edited (GE) CytoRP plants express a PRORP2 mutant, where 16 amino-acids (aa) are deleted and the 10 first aa of PRORP2 are in frame with aa 27 to 528. Deleted aa contain the 4 lysine residues that contribute to wild type PRORP2 nuclear localization. PCR genotyping presented here in b and c was performed a single time. However, the genotype of mutant plants was systematically verified and confirmed by PCR before each virus infection experiment.

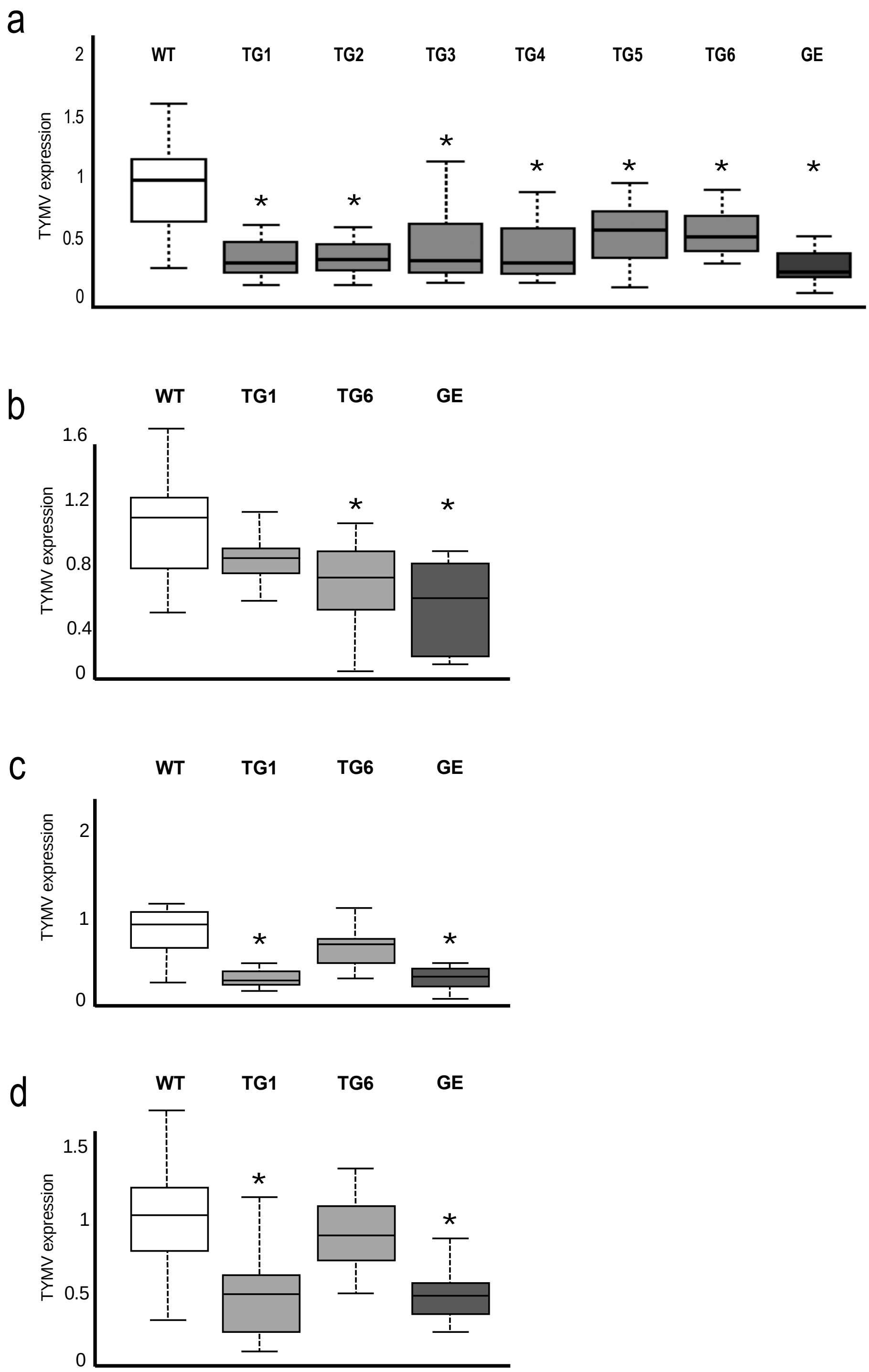

Supplementary Figure 5 Quantification of TYMV RNA levels accumulating in wild type plants (WT) as well as in transgenic CytoRP lines (TG) and a cisgenic line obtained by genome editing using CRISPR Cas9 technology (GE). TG1 to TG4 as well as GE use PRORP2 endogenous promoter while in TG5 and 6 lines, CytoRP is expressed under the control of the CaMV 35S promoter. 10 to 20 plants of each lines were inoculated with TYMV RNA in different sets of experiments (a-d) and the accumulation of viral RNA progeny was determined by RTqPCR. The results, normalized to the mean value of WT plants in each experiment, are represented as box plots, with the thick black line representing the median value and boxes representing the first and third quartiles. Statistically significant differences of virus levels in CytoRP lines as compared to WT were assessed by Wilcoxon tests and indicated by stars.

Source data are provided as a Source Data file.

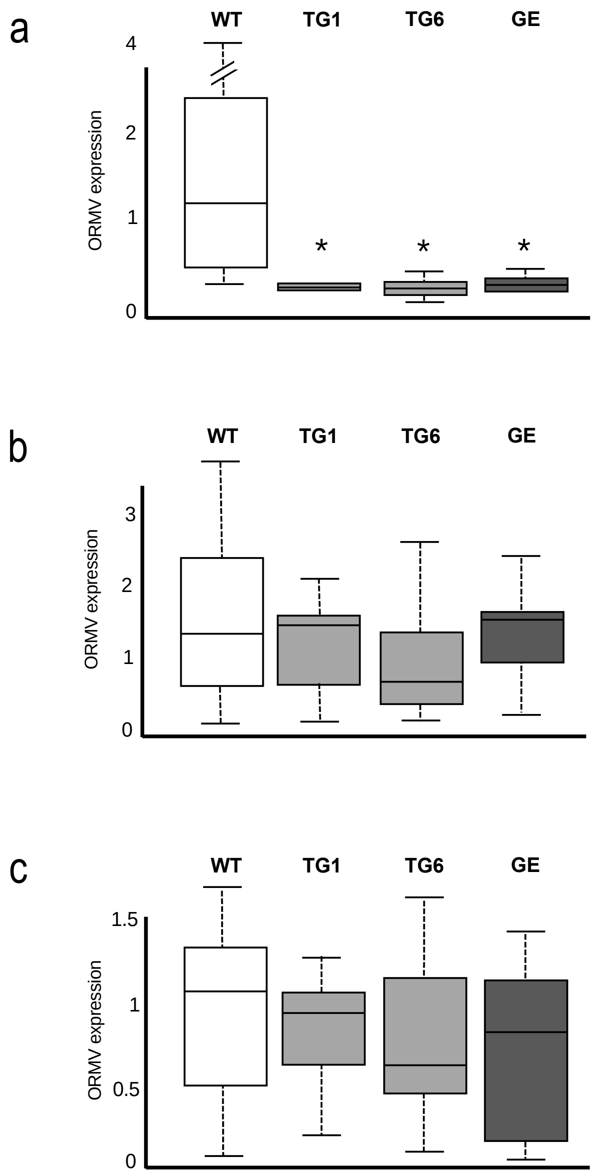

Supplementary Figure 6 Quantification of ORMV RNA levels accumulating in WT plants as well as in transgenic CytoRP lines expressing CytoRP with PRORP2 endogenous promoter (TG1) , expressing CytoRP under the control of the CaMV 35S promoter (TG6) and a cisgenic line obtained by genome editing using CRISPR Cas9 technology (GE). The accumulation of viral RNA progeny was determined by RTqPCR in different sets of experiments (a-c), in which, at least 10 plants from each independent line were inoculated with ORMV. The results, normalized to the mean value of WT plants in each experiment, are represented as box plots, with the black line representing the median value and boxes representing the first and third quartiles. Statistically significant differences of virus levels in CytoRP lines as compared to WT were assessed by Wilcoxon tests and indicated by stars.

Source data are provided as a Source Data file.

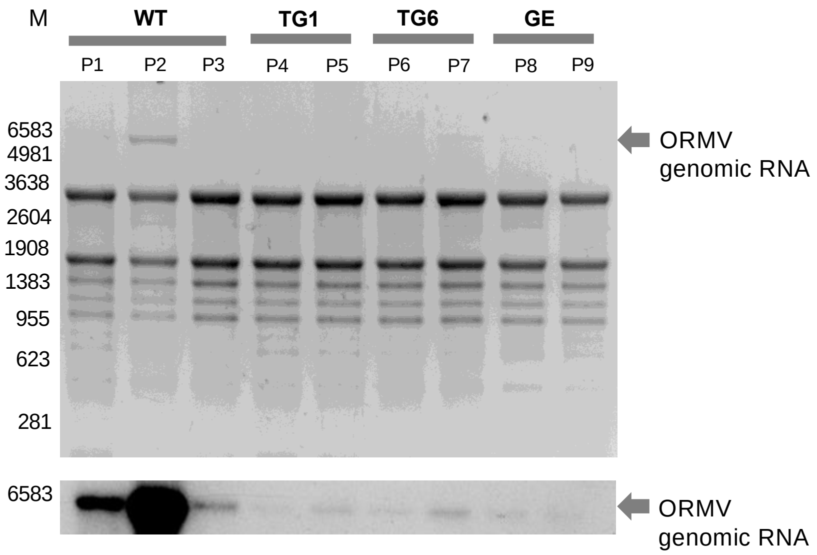

Supplementary Figure 7 Quantification of ORMV RNA levels accumulating in wild type plants (WT) as well as in transgenic CytoRP lines TG1 with endogenous promoter, TG6 with the CaMV 35S promoter and the cisgenic line GE obtained by genome editing. ORMV levels of the experiments presented in Supplementary Figure 6a were also monitored by RNA gel analysis. Total RNAs from 3 representative wild type plants (P1-P3), 2 plants from TG1, TG6 and GE lines (P4-P9) infected by ORMV were separated a 1% denaturing agarose gel (top panel), transferred to a membrane and hybridized with an ORMV RNA specific probe (bottom panel) revealing the 6 kbp ORMV genomic RNA, indicated by grey arrows. This experiment was performed two times.

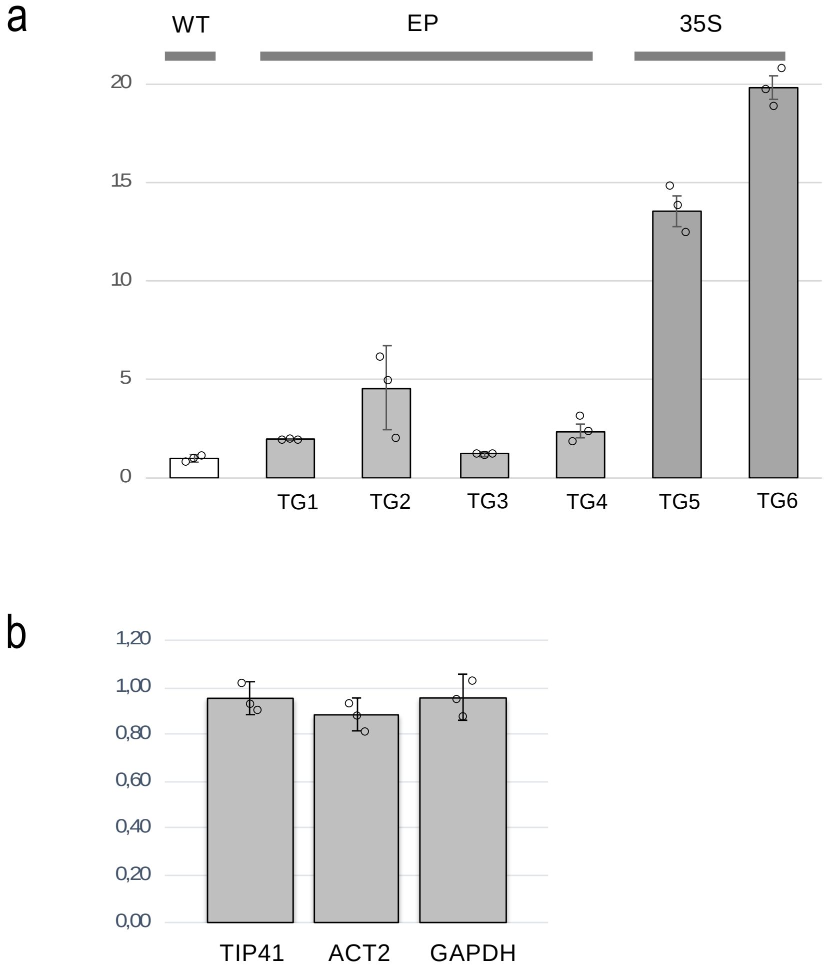

Supplementary Figure 8 Expression of CytoRP mRNA relative to that of endogenous PRORP2 measured by quantitative RT-PCR. **(a)** Expression of CytoRP was measured for the 6 transgenic lines expressing CytoRP (TG1 to TG6) under the control of PRORP2 endogenous promoter (EP) or the CaMV 35S promoter (35S). Bars represent the mean values of 3 replicate experiments and error bars indicate standard deviations. Individual data points are represented by dots. TIP41, ACT2 and GAPDH were used as reference genes. **(b)** The relative expression level of CytoRP in the genome edited line compared to wild type PRORP2 is presented using either TIP41, ACT2 or GAPDH as reference genes. Values close to 1 indicate that both genes have similar expression levels. Bars represent the mean values of 3 replicate experiments and error bars indicate standard deviations. Individual data points are represented by dots.

Supplementary Table 1. List of plant viruses holding tRNA-like structures (TLS), for which aminoacylation could be established.

Genus	*Virus*	*Accessions*
Tymovirus	Turnip yellow mosaic virus (TYMV)	NC_004063.1
	Andean potato latent virus (APLV)	NC_020470.1
	Belladonna mottle virus (BeMV)	NC_038866.1 partial
	Cacao yellow mosaic virus (CYMV)	NC_038867.1 partial
	Clitoria yellow vein virus (CYVV)	M58311.1 (TLS)
	Eggplant mosaic virus (EMV)	NC_001480.1
	Kennedya yellow mosaic virus (KYMV)	NC_001746.1
	Okra mosaic virus (OkMV)	NC_009532.1
	Ononis yellow mosaic virus (OYMV)	NC_001513.1
	Wild cucumber mosaic virus (WCMV)	AF035633 partial
	Nemesia ring necrosis virus (NeRNV)	NC_011538.1
Furovirus	Soil-borne wheat mosaic virus (SBWMV)	NC_002041.1 , NC_002042.1
Pomovirus	Beet soil-borne virus (BSBV)	NC_003520.1 , NC_003518.1 , NC_003519.1
	Potato mop-top virus (PMTV)	NC_003723.1 , NC_003725.1 , NC_003724.1
Pecluvirus	Indian peanut clump virus (IPCV)	NC_004729.1 , NC_004730.1
	Peanut clump virus (PCV)	NC_003672.1 , NC_003668.1
Tobamovirus	Tobacco mosaic virus (TMV)	NC_001367.1
	Oilseed rape mosaic virus (ORMV)	KF137561
	Cucumber green mottle mosaic virus (CGMMV)	NC_001801.1
	Green tomato atypical mosaic virus (GTAMV)	-
	Satellite tobacco mosaic virus (STMV)	NC_001557.1
	Sunnhemp mosaic virus (SHMV)	U47034.1 , J02413.1 partial
Bromovirus	Brome mosaic virus (BMV)	NC_002026.1 , NC_002027.1 , NC_002028.2
	Broad bean mottle virus (BBMV)	NC_004008.1 , NC_004007.1 , NC_004006.1
	Cowpea chlorotic mottle virus (CCMV)	NC_003543.1 , NC_003541.1 , NC_003542.1
Cucumovirus	Cucumber mosaic virus (CMV)	NC_002034.1 , NC_002035.1 , NC_001440.1
Hordeivirus	Barley stripe mosaic virus (BSMV)	NC_003469.1 , NC_003481.1 , NC_003478.1
	Poa semilatent virus (PSLV)	M81486.1 , Z46352.1 , M81487.1 partial

TLS containing viruses represent a wide distribution of virus genera. Accessions refer to NCBI database. Virus names were often attributed according to the first plants in which the respective viruses were found and thus do not reflect their complete host range. For example, the *Cucumber mosaic virus* (CMV) infects over 1000 plant species (especially tomato, banana, pepper, cucurbits and legumes) and is thus of major economic importance.

Supplementary Table 2 List of DNA primers used in this study. Names of oligonucleotide are indicated in bold and their sequences are given from 5’ to 3’.

**CRP5’**	ATGGTAAGCAGAAACCCAGAAAC
**CRP3’**	AGGAATCTTCCCATTACTCTT
**TYMV5’**	ATGGATCCGAATTGTAATACGACTCACTATAGCCTAAGTTCTCGATCTTTAAAATCG
**TYMV3’**	ATGAATTCTGGTTCCGATGACCCTCGGAAG
**ORMV5’**	GAATTGTAATACGACTCACTATAGGGTTATTTCCTGGATCACCTG
**ORMV3’**	TGGGCCCCTACCCGGG
**CRTTYMV5’**	TCTGTCCCCACACGACAGATAAT
**CRTTYMV3’**	GCGAGCTAACGATTTTAAAGATCG
**CRTORMV5’**	GTTATTTCCTGGATCACCTGTTAACGTACGC
**CRTORMV3’**	CCTTGGCACCCGAGAATTCCA
**GtTg5’**	TGCTTGTGACAAATGATGAG
**GtTg3’**	AGCGTAATCTGGAACATCGTATGG
**GtCg5’**	GGTTTAGGGTTTCATCTACCAC
**GtCg3’**	CACGGTCAATGGCAAGGTATT
