# Supplementary material for: Towards plant resistance to viruses using protein-only RNase P
Source: Nat Commun. 2021 Feb 12;12:1007. doi: 10.1038/s41467-021-21338-6 (PMC7881203; doi:10.1038/s41467-021-21338-6)
Supplement: Supplementary file 2 — Reporting Summary [file 41467_2021_21338_MOESM2_ESM.pdf]

nature research

Corresponding author(s): Anthony Gobert
Isabelle Jupin
Philippe Giegé

Last updated by author(s): Jan 21, 2021

Reporting Summary

Nature Research wishes to improve the reproducibility of the work that we publish. This form provides structure for consistency and transparency in reporting. For further information on Nature Research policies, see our Editorial Policies and the Editorial Policy Checklist.

Statistics

For all statistical analyses, confirm that the following items are present in the figure legend, table legend, main text, or Methods section.

n/a	Confirmed	
☐	☒	The exact sample size (n) for each experimental group/condition, given as a discrete number and unit of measurement
☐	☒	A statement on whether measurements were taken from distinct samples or whether the same sample was measured repeatedly
☐	☒	The statistical test(s) used AND whether they are one- or two-sided<br>*Only common tests should be described solely by name; describe more complex techniques in the Methods section.*
☒	☐	A description of all covariates tested
☐	☒	A description of any assumptions or corrections, such as tests of normality and adjustment for multiple comparisons
☐	☒	A full description of the statistical parameters including central tendency (e.g. means) or other basic estimates (e.g. regression coefficient) AND variation (e.g. standard deviation) or associated estimates of uncertainty (e.g. confidence intervals)
☐	☒	For null hypothesis testing, the test statistic (e.g. F, t, r) with confidence intervals, effect sizes, degrees of freedom and P value noted<br>*Give P values as exact values whenever suitable.*
☒	☐	For Bayesian analysis, information on the choice of priors and Markov chain Monte Carlo settings
☒	☐	For hierarchical and complex designs, identification of the appropriate level for tests and full reporting of outcomes
☒	☐	Estimates of effect sizes (e.g. Cohen's d, Pearson's r), indicating how they were calculated

Our web collection on statistics for biologists contains articles on many of the points above.

Software and code

Policy information about availability of computer code

Data collection	-For localisation experiments, eYFP fluorescencen, Mitotracker and DAPI staining of protoplasts were observed by confocal laser scanning microscopy using a Zeiss LSM700 based on an Axiovert 200M microscope (Zeiss).<br>-For RNA cleavage assays, signals were acquired either by autoradiography and / or with a FLA-7000 phosphorimager (Fujifilm).<br>-For viral RNA level quantifications, qRT-PCR data were acquired by Bio-Rad CFX96 qPCR machine and by a Roche LightCycler 480 machine.
Data analysis	qRT-PCR experiments were analysed with the LightCycler 480 software v4.1 (Roche). The results, normalized to the mean value of WT plants in each experiment, were represented as box plots, with the thick black line representing the median value and boxes representing the first and third quartiles.<br>PyMol v2.0.7 was used to display a model of CytoRP interaction with the TYMV TLS.<br>MUSCLE alignment v2019 was used to compare PRORP sequences and define conserved features.<br>SUBA v4 was used to determine subcellular localization predictions.<br>Phyre2 v2.0 was used to perform protein 3D structure predictions.

For manuscripts utilizing custom algorithms or software that are central to the research but not yet described in published literature, software must be made available to editors and reviewers. We strongly encourage code deposition in a community repository (e.g. GitHub). See the Nature Research guidelines for submitting code & software for further information.

Data

Policy information about availability of data

All manuscripts must include a data availability statement. This statement should provide the following information, where applicable:

- Accession codes, unique identifiers, or web links for publicly available datasets
- A list of figures that have associated raw data
- A description of any restrictions on data availability

Data supporting the findings of this work are available within the paper and its Supplementary Information files. The source data underlying Figures 1 and 2, as well as Supplementary Figures 3, 5, and 6 are provided as a Source Data file.

Field-specific reporting

Please select the one below that is the best fit for your research. If you are not sure, read the appropriate sections before making your selection.

[x] Life sciences [ ] Behavioural & social sciences [ ] Ecological, evolutionary & environmental sciences

For a reference copy of the document with all sections, see nature.com/documents/nr-reporting-summary-flat.pdf

Life sciences study design

All studies must disclose on these points even when the disclosure is negative.

Sample size	-For biochemical studies, kinetics were performed in tripllicate experiments, with 10 different time points and 4 different substrate RNA concentrations. Similar sample sizes are used in the litereature for similar studies e.g. Gutmann et al. 2012 (doi:10.1101/gad.189514.112).<br>-Altogether virus infections were analysed in 7 sets of independant experiments. In each set of experiments >10 individual plants were analysed for each genotype, with 4 to 8 genotypes analysed in one set of experiments. Similar sample sizes are used in the litereature for similar studies e.g. Moriceau et al. 2017 (doi.org/10.3389/fpls.2017.02138).
Data exclusions	No data exclusion.
Replication	-Kinetic experiments were perfomed in 3 independent replicates for each condition (individual time point and RNA concentration). All replication of RNase P cleavage assays were successful.<br>-For qRT-PCR analyses, for each set of experiments, samples from at least 10 independently grown plants were analysed for each genotype. 3 technical repeats were performed for each individual plant sample. While technical replicates were always successful, individual sets of experiments gave variable results as shown on Supplementary Figures 5 and 6.
Randomization	Plants were all grown in the same standard conditions (see Methods) and were placed randomly in the growth room.
Blinding	For each genotype, plants were randomly selected. After virus infection, all the leaves that had appeared 28 and 7 days post infection for TYMV and ORMV infections respectively were collected for analysis. Blinding for leaf collection was thus not necessary.

Reporting for specific materials, systems and methods

We require information from authors about some types of materials, experimental systems and methods used in many studies. Here, indicate whether each material, system or method listed is relevant to your study. If you are not sure if a list item applies to your research, read the appropriate section before selecting a response.

Materials & experimental systems

n/a	Involved in the study	
[x]	[ ]	Antibodies
[x]	[ ]	Eukaryotic cell lines
[x]	[ ]	Palaeontology and archaeology
[x]	[ ]	Animals and other organisms
[x]	[ ]	Human research participants
[x]	[ ]	Clinical data
[x]	[ ]	Dual use research of concern

Methods

n/a	Involved in the study	
[x]	[ ]	ChIP-seq
[x]	[ ]	Flow cytometry
[x]	[ ]	MRI-based neuroimaging
