# Supplementary material for: Towards plant resistance to viruses using protein-only RNase P
Source: Nat Commun. 2021 Feb 12;12:1007. doi: 10.1038/s41467-021-21338-6 (PMC7881203; doi:10.1038/s41467-021-21338-6)
Supplement: Supplementary file 3 — Source Data [file 41467_2021_21338_MOESM3_ESM.zip › Source data Fig 2d.pptx]

## Slide 1
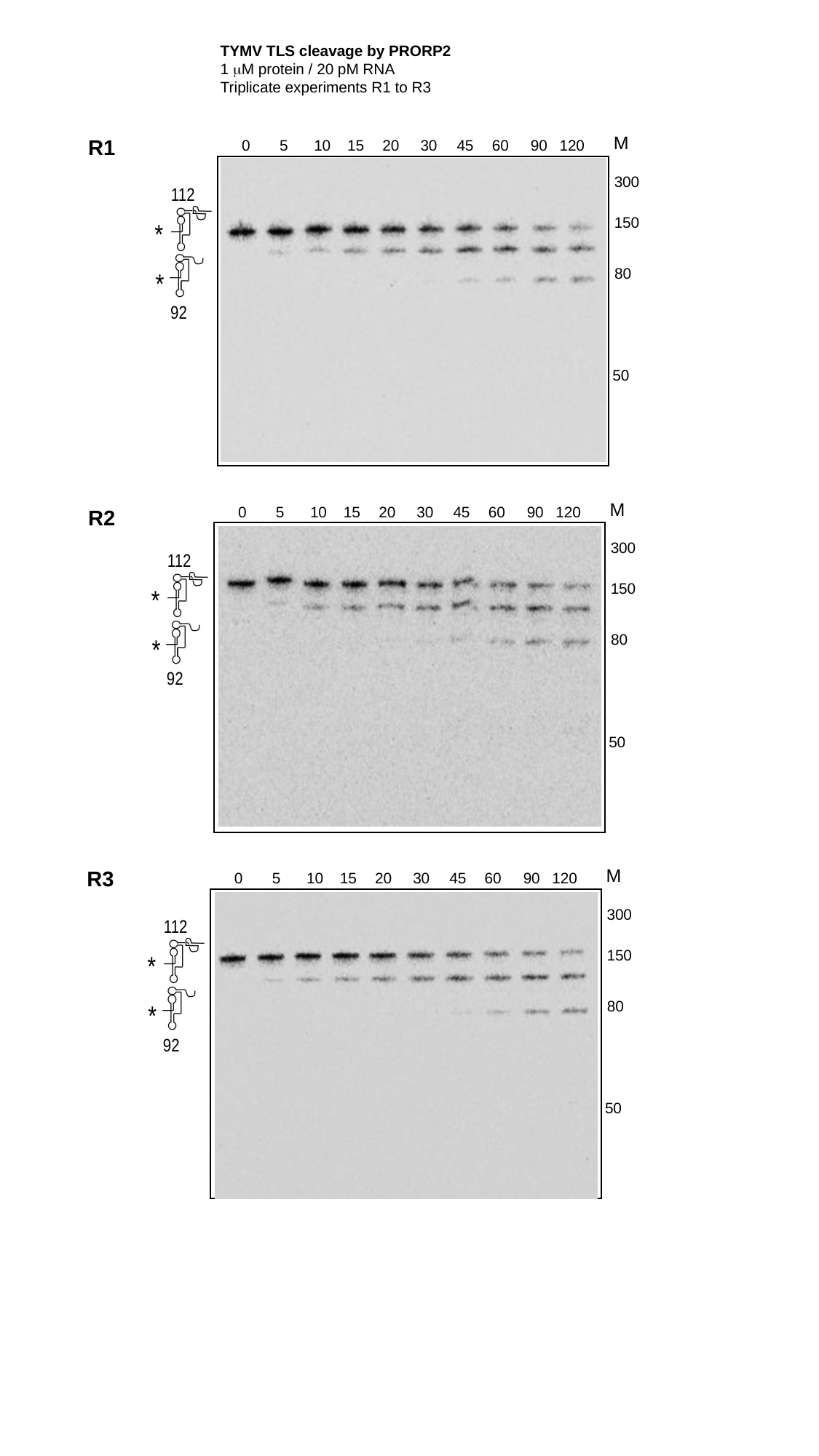

TYMV TLS cleavage by PRORP2
1 mM protein / 20 pM RNA
Triplicate experiments R1 to R3
M
0
5
10
15
20
30
45
60
90
120
300
112
*
*
92
150
80
50
R1
M
0
5
10
15
20
30
45
60
90
120
300
112
*
*
92
150
80
50
R2
M
0
5
10
15
20
30
45
60
90
120
300
112
*
*
92
150
80
50
R3

## Slide 2
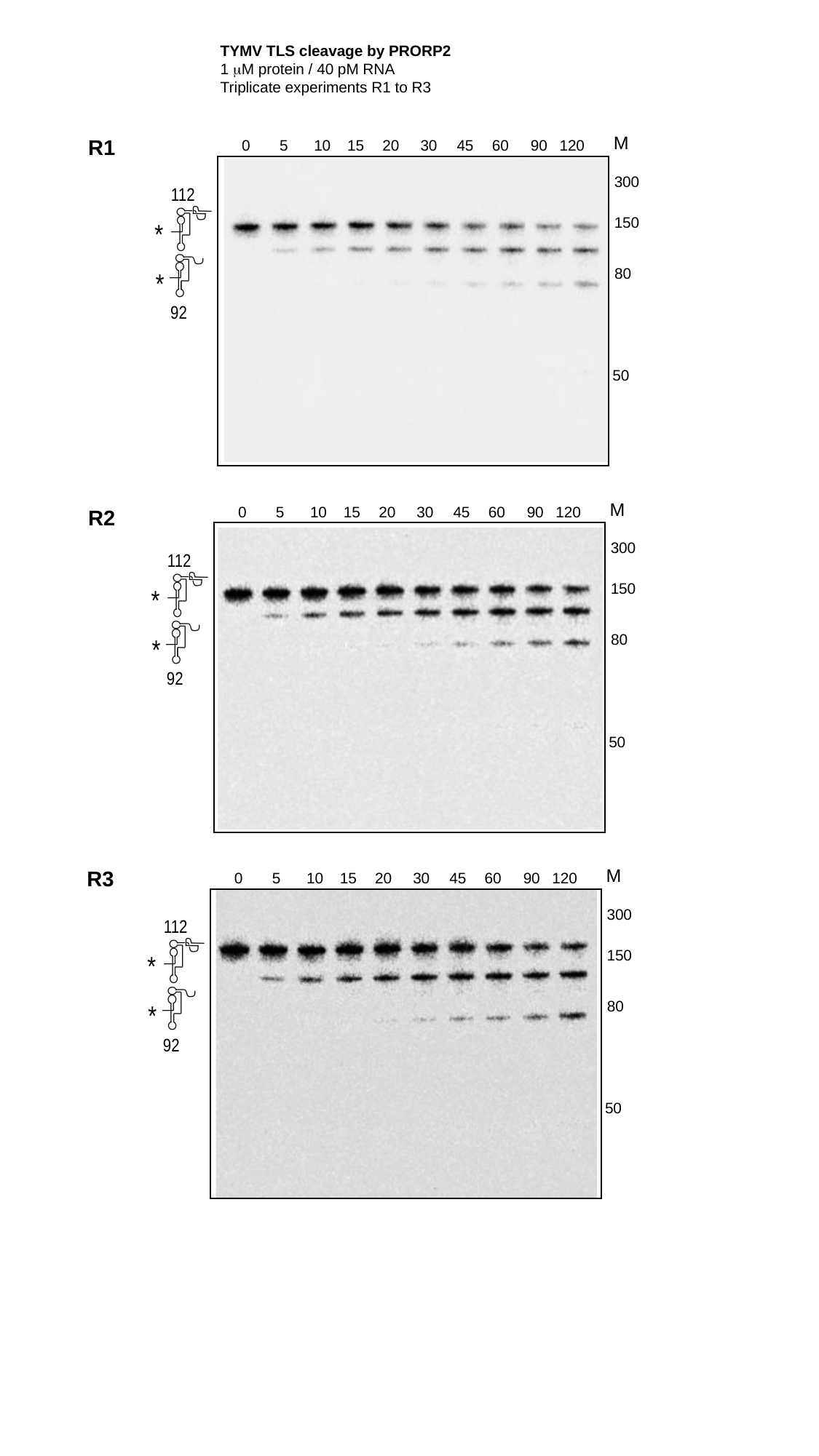

TYMV TLS cleavage by PRORP2
1 mM protein / 40 pM RNA
Triplicate experiments R1 to R3
M
0
5
10
15
20
30
45
60
90
120
300
112
*
*
92
150
80
50
R1
M
0
5
10
15
20
30
45
60
90
120
300
112
*
*
92
150
80
50
R2
M
0
5
10
15
20
30
45
60
90
120
300
112
*
*
92
150
80
50
R3

## Slide 3
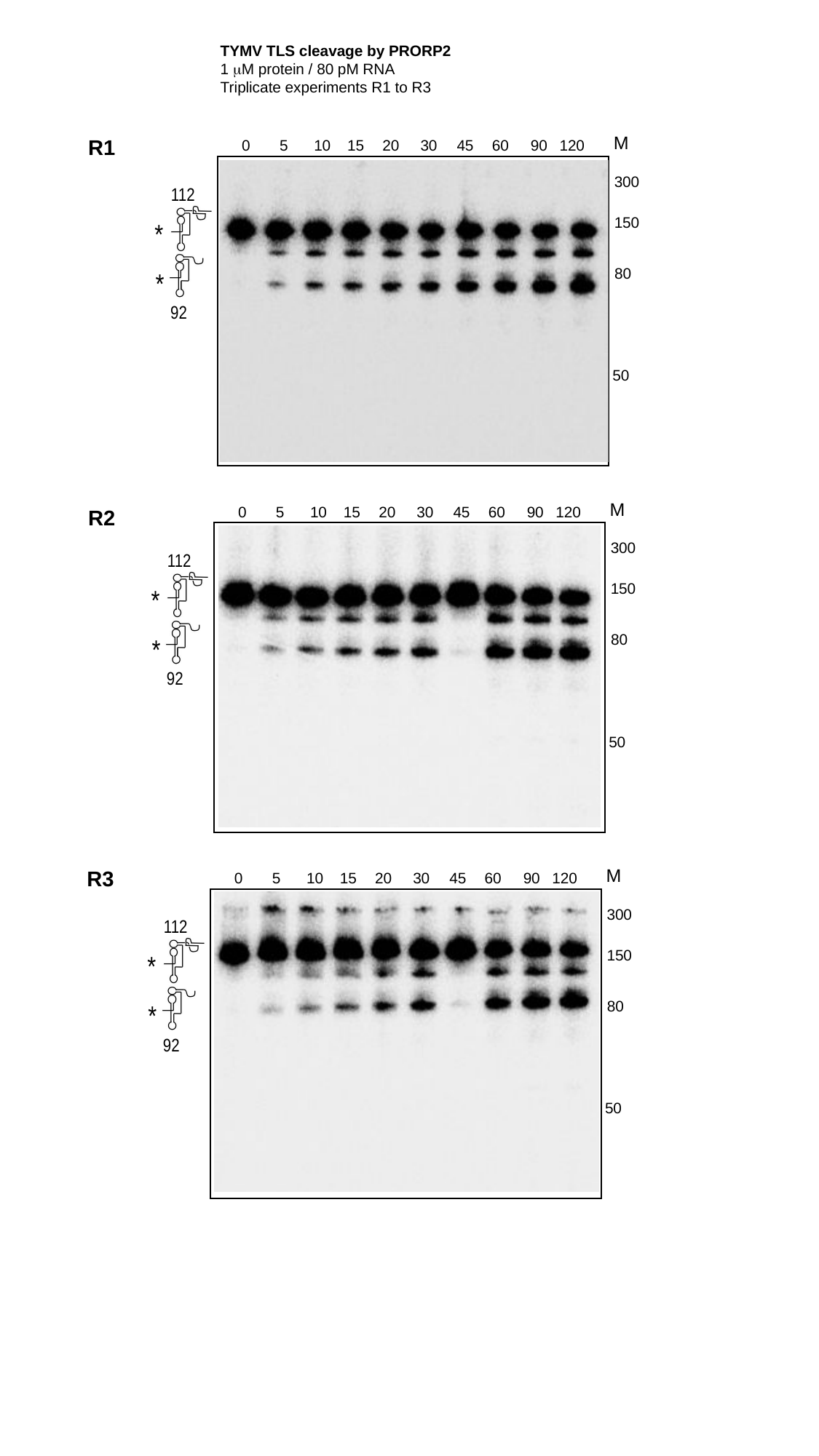

TYMV TLS cleavage by PRORP2
1 mM protein / 80 pM RNA
Triplicate experiments R1 to R3
M
0
5
10
15
20
30
45
60
90
120
300
112
*
*
92
150
80
50
R1
M
0
5
10
15
20
30
45
60
90
120
300
112
*
*
92
150
80
50
R2
M
0
5
10
15
20
30
45
60
90
120
300
112
*
*
92
150
80
50
R3

## Slide 4
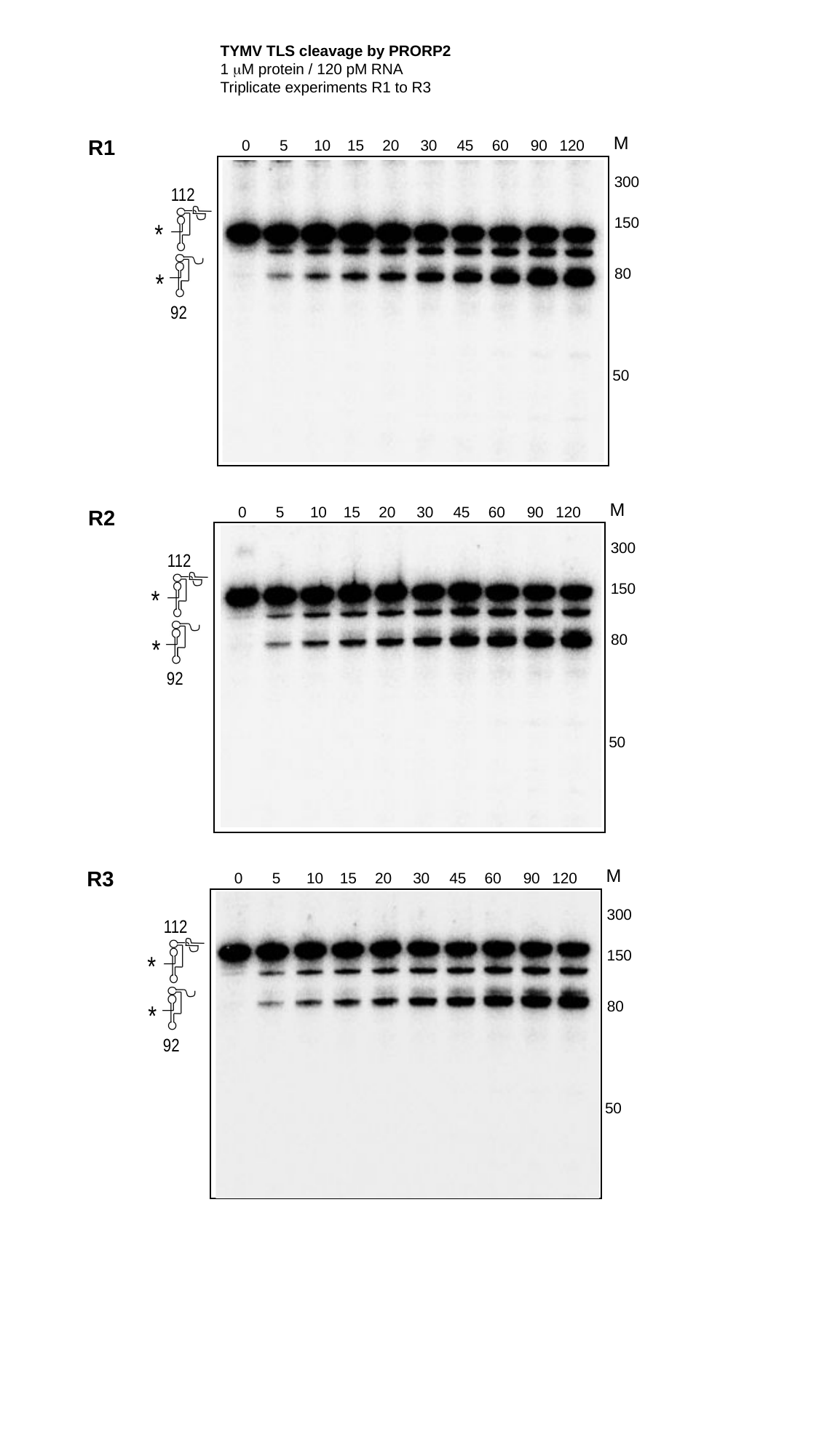

TYMV TLS cleavage by PRORP2
1 mM protein / 120 pM RNA
Triplicate experiments R1 to R3
M
0
5
10
15
20
30
45
60
90
120
300
112
*
*
92
150
80
50
R1
M
0
5
10
15
20
30
45
60
90
120
300
112
*
*
92
150
80
50
R2
M
0
5
10
15
20
30
45
60
90
120
300
112
*
*
92
150
80
50
R3

## Slide 5
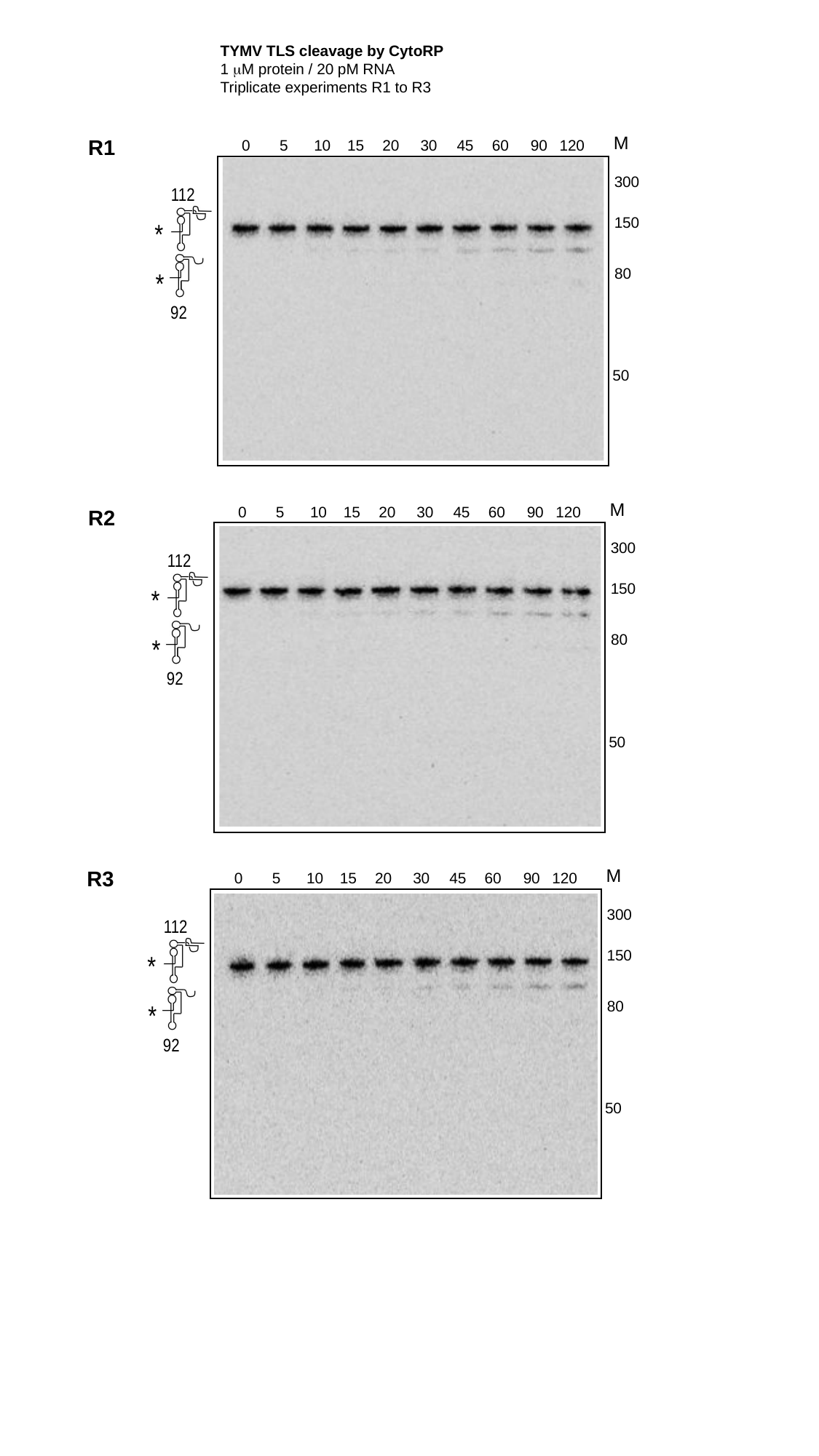

TYMV TLS cleavage by CytoRP
1 mM protein / 20 pM RNA
Triplicate experiments R1 to R3
M
0
5
10
15
20
30
45
60
90
120
300
112
*
*
92
150
80
50
R1
M
0
5
10
15
20
30
45
60
90
120
300
112
*
*
92
150
80
50
R2
M
0
5
10
15
20
30
45
60
90
120
300
112
*
*
92
150
80
50
R3

## Slide 6
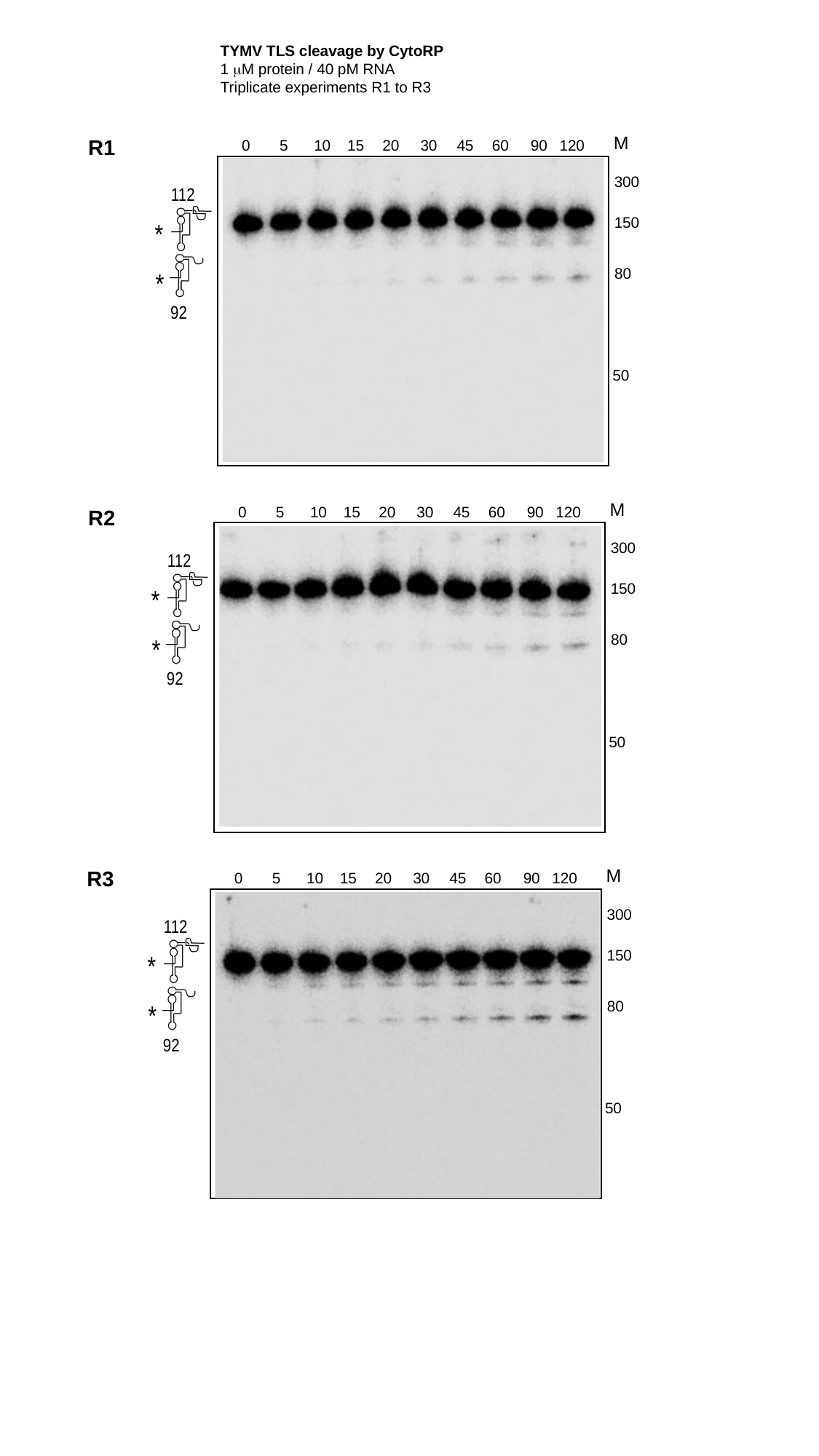

TYMV TLS cleavage by CytoRP
1 mM protein / 40 pM RNA
Triplicate experiments R1 to R3
M
0
5
10
15
20
30
45
60
90
120
300
112
*
*
92
150
80
50
R1
M
0
5
10
15
20
30
45
60
90
120
300
112
*
*
92
150
80
50
R2
M
0
5
10
15
20
30
45
60
90
120
300
112
*
*
92
150
80
50
R3

## Slide 7
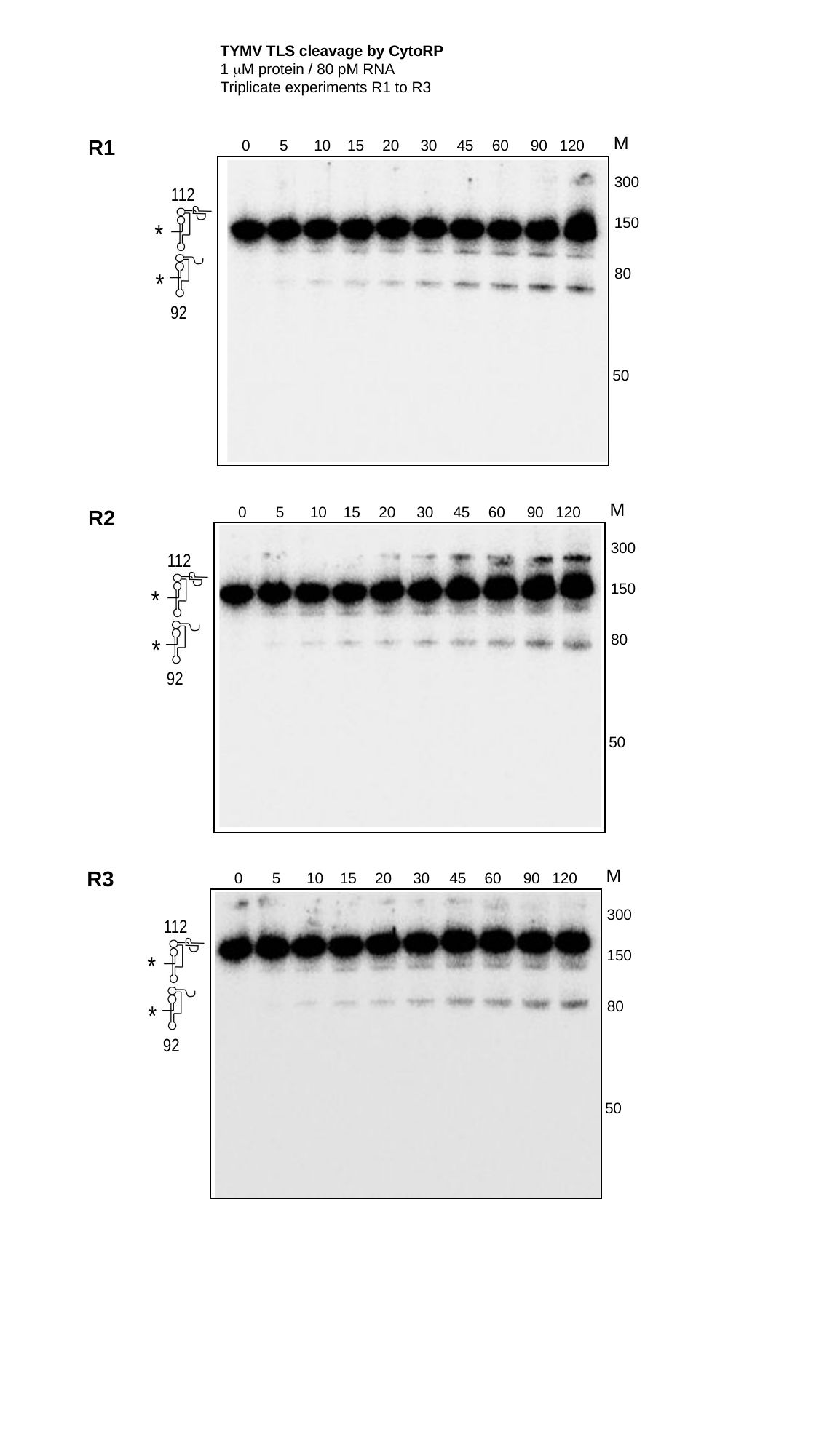

TYMV TLS cleavage by CytoRP
1 mM protein / 80 pM RNA
Triplicate experiments R1 to R3
M
0
5
10
15
20
30
45
60
90
120
300
112
*
*
92
150
80
50
R1
M
0
5
10
15
20
30
45
60
90
120
300
112
*
*
92
150
80
50
R2
M
0
5
10
15
20
30
45
60
90
120
300
112
*
*
92
150
80
50
R3

## Slide 8
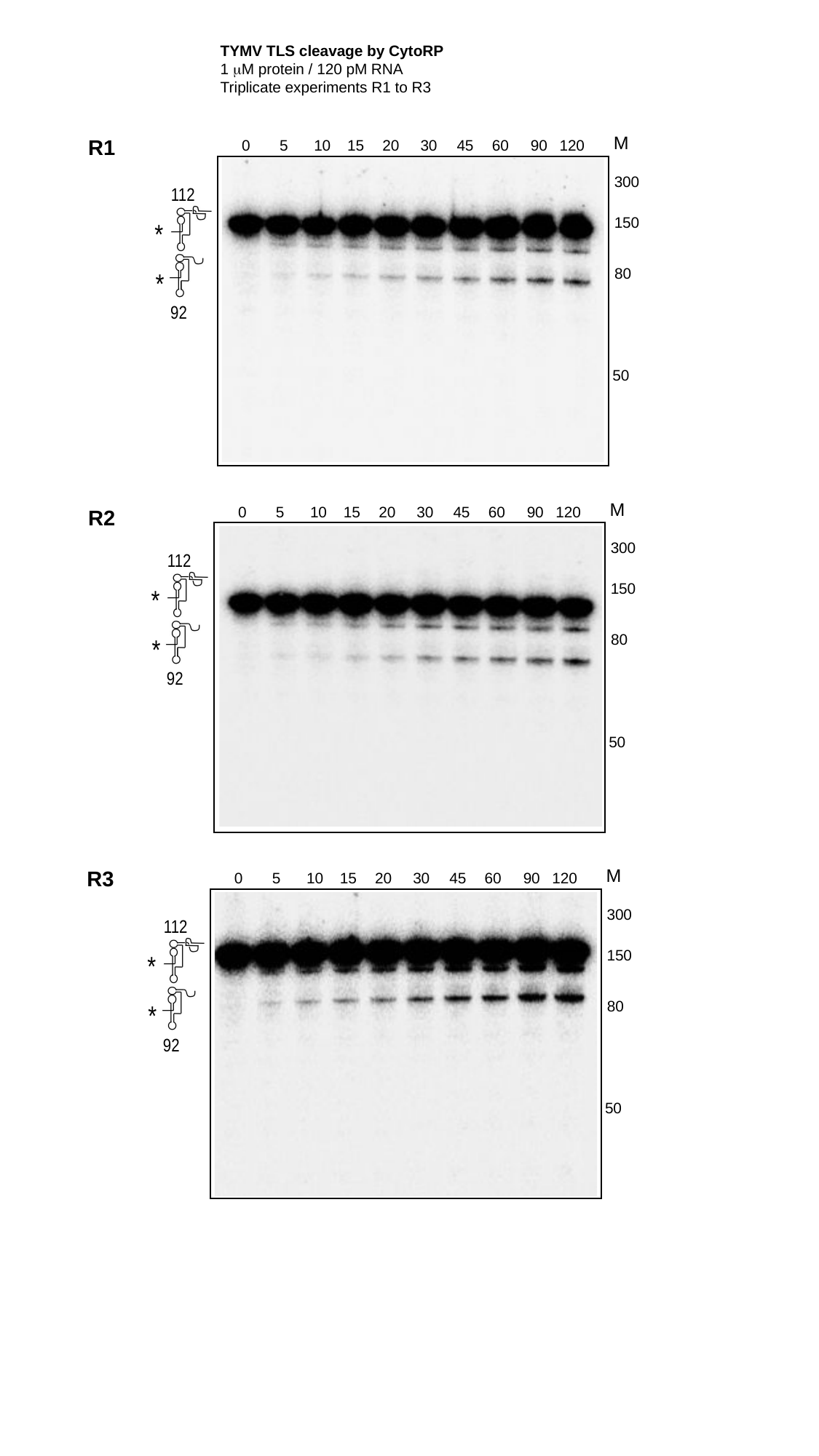

TYMV TLS cleavage by CytoRP
1 mM protein / 120 pM RNA
Triplicate experiments R1 to R3
M
0
5
10
15
20
30
45
60
90
120
300
112
*
*
92
150
80
50
R1
M
0
5
10
15
20
30
45
60
90
120
300
112
*
*
92
150
80
50
R2
M
0
5
10
15
20
30
45
60
90
120
300
112
*
*
92
150
80
50
R3
