# Supplementary figures and images for: Towards plant resistance to viruses using protein-only RNase P
Source: Nat Commun. 2021 Feb 12;12:1007. doi: 10.1038/s41467-021-21338-6 (PMC7881203; doi:10.1038/s41467-021-21338-6)

## Slide 1
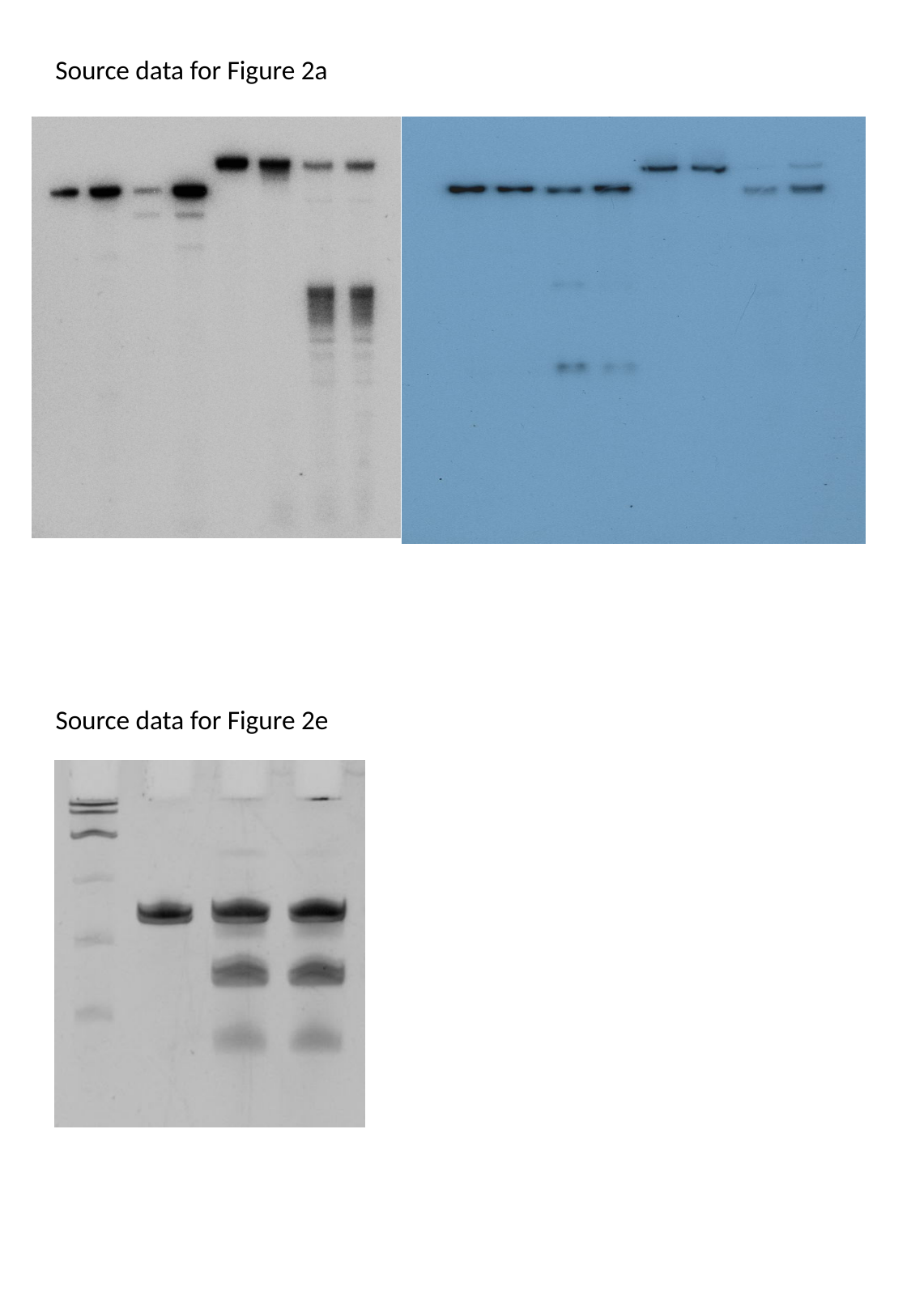

Source data for Figure 2a
Source data for Figure 2e

Supplement: Supplementary file 3 — Source Data [file 41467_2021_21338_MOESM3_ESM.zip › Source data Fig 2a and e.pptx]

## Slide 1
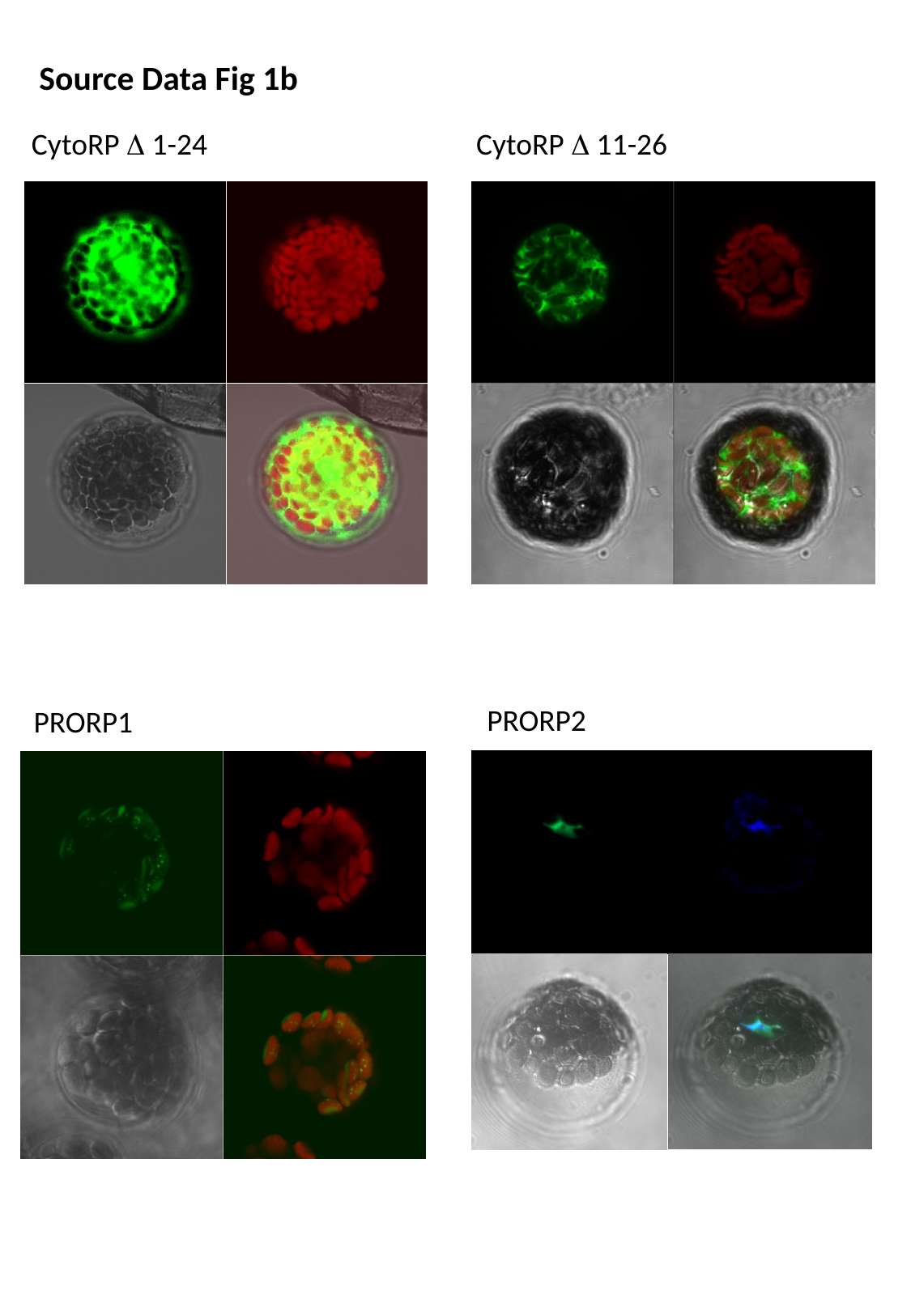

Source Data Fig 1b
CytoRP D 1-24
CytoRP D 11-26
PRORP2
PRORP1

Supplement: Supplementary file 3 — Source Data [file 41467_2021_21338_MOESM3_ESM.zip › Source data Fig 1b.pptx]
